# Associations between cardiac resynchronization therapy and clinical outcomes according to the atrial fibrillation status in patients with heart failure with reduced ejection fraction

**DOI:** 10.1093/europace/euaf296

**Published:** 2025-11-18

**Authors:** Renzo Laborante, Valeria Valente, Lina Benson, Paolo Gatti, Christian Basile, Alessandro Villaschi, Peter Moritz Becher, Domenico D’Amario, Carin Corovic-Cabrera, Fredrik Gadler, Gianluigi Savarese, Raffaele Scorza

**Affiliations:** Department of Clinical Science and Education, Södersjukhuset, Karolinska Institutet, Sjukhusbacken 10, 118 83, Stockholm, Sweden; Department of Clinical Science and Education, Södersjukhuset, Karolinska Institutet, Sjukhusbacken 10, 118 83, Stockholm, Sweden; Department of Clinical Science and Education, Södersjukhuset, Karolinska Institutet, Sjukhusbacken 10, 118 83, Stockholm, Sweden; Division of Cardiology, Department of Medicine, Karolinska Institutet, Eugeniavägen 27, 171 64, Stockholm, Sweden; Division of Cardiology, Department of Medicine, Karolinska Institutet, Eugeniavägen 27, 171 64, Stockholm, Sweden; Department of Clinical Science and Education, Södersjukhuset, Karolinska Institutet, Sjukhusbacken 10, 118 83, Stockholm, Sweden; National Association of Hospital Cardiologists (ANMCO) Research Center, Heart Care Foundation, Via Alfonso La Marmora, 36, 50121, Florence, Italy; Department of Clinical Science and Education, Södersjukhuset, Karolinska Institutet, Sjukhusbacken 10, 118 83, Stockholm, Sweden; Department of Biomedical Sciences, Humanitas University, Pieve Emanuele, Via Rita Levi Montalcini 4, 20090, Milan, Italy; De Gasperis Cardio Center, Niguarda Hospital, Piazza Ospedale Maggiore 3, 20162, Milan, Italy; Department of Clinical Science and Education, Södersjukhuset, Karolinska Institutet, Sjukhusbacken 10, 118 83, Stockholm, Sweden; Department of Cardiology, University Heart and Vascular Center Hamburg, Martinistraße 52, 20246, Hamburg, Germany; German Center of Cardiovascular Research (DZHK), Partner Site Hamburg/Kiel/Lübeck, Martinistraße 52, 20246, Hamburg, Germany; Department of Translational Medicine, University of Eastern Piedmont, Via Solaroli 17, 28100, Novara, Italy; Thoraco-Cardio-Vascular Department, Azienda Ospedaliero-Universitaria Maggiore della Carità, Largo Bellini 15, 28100, Novara, Italy; Department of Clinical Science and Education, Södersjukhuset, Karolinska Institutet, Sjukhusbacken 10, 118 83, Stockholm, Sweden; Division of Cardiology, Department of Medicine, Karolinska Institutet, Eugeniavägen 27, 171 64, Stockholm, Sweden; Heart, Vascular and Neuro Theme, Karolinska University Hospital, Eugeniavägen 27, 171 64, Stockholm, Sweden; Department of Clinical Science and Education, Södersjukhuset, Karolinska Institutet, Sjukhusbacken 10, 118 83, Stockholm, Sweden; Department of Clinical Science and Education, Södersjukhuset, Karolinska Institutet, Sjukhusbacken 10, 118 83, Stockholm, Sweden

**Keywords:** Heart failure, Cardiac resynchronization therapy, Atrial fibrillation

## Abstract

**Aims:**

To evaluate in patients with heart failure with reduced ejection fraction (HFrEF) the association between patient characteristics and likelihood of receiving cardiac resynchronization therapy (CRT), as well as between CRT and clinical outcomes, according to comorbid atrial fibrillation (AF).

**Methods and results:**

Patients in the Swedish Heart Failure (HF) Registry who met the guidelines’ recommendation for CRT between 2014 and 2022 were included. The primary endpoint was the composite of time to first HF hospitalization or cardiovascular (CV) death. Secondary endpoints were its individual components, all-cause death, and the total number of HF hospitalizations. Out of 3530 patients with HFrEF and an indication for CRT, 24.7% received a CRT. A history of or concomitant AF were observed in 51.6% of patients. AF was not associated with the likelihood of receiving a CRT, and the patient characteristics independently associated with CRT were consistent regardless of AF, except for CRT being less likely implanted in patients with valvular disease without AF, and more likely among those with AF and university (vs. compulsory) education. Regardless of AF, CRT use was associated with a lower adjusted risk of CV death/first HF hospitalization [hazard ratio (HR): 0.71, 95% confidence interval (CI) 0.64–0.79], of its individual components, and of all-cause death (HR: 0.72, 95% CI 0.64–0.81), but not with total number of HF hospitalizations.

**Conclusion:**

A diagnosis of AF was not associated with the likelihood of receiving CRT in real-world HF care, nor did it affect the association between CRT and lower risk of clinical outcomes.

## Graphical Abstract

**Graphical Abstract:**
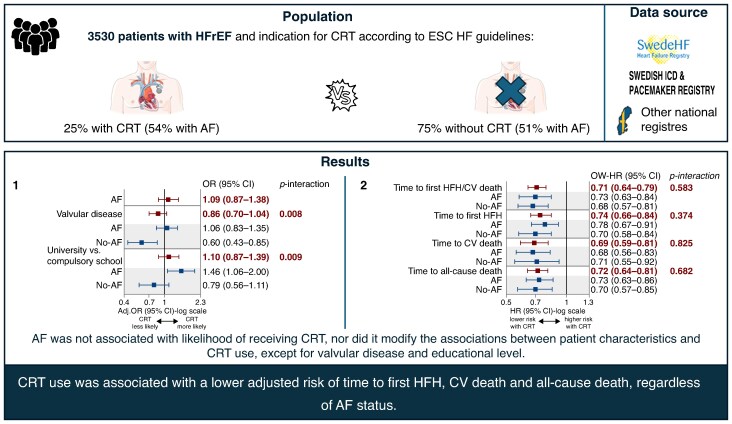
Out of 3530 patients with HFrEF and an indication for CRT, 24.7% received a CRT. AF was not associated with the likelihood of receiving a CRT and did not modify the associations between patient characteristics and CRT use, except for CRT being less likely to be implanted in patients with valvular disease without AF, and more likely among those with AF and university (vs. compulsory) education. CRT was associated with reduced adjusted risks of first HFH, CV death, and all-cause death, regardless of AF status. Adj, adjusted; AF, atrial fibrillation; CI, confidence interval; CRT, cardiac resynchronization therapy; CV, cardiovascular; ESC, European Society of Cardiology; HF, heart failure; HFH, heart failure hospitalization; HFrEF, heart failure with reduced ejection fraction; HR, hazard ratio; OR, odds ratio; OW, overlap weighting.

What’s new?

- In a nationwide real-world cohort of patients with HFrEF fulfilling guideline criteria for CRT implantation, AF did not influence the likelihood of receiving CRT.
- Factors associated with the likelihood of CRT implantation were largely consistent across AF status, except for CRT being less likely to be implanted in patients with valvular disease without AF, and more likely among those with AF and university (vs. compulsory) education.
- CRT use was associated with a lower risk of CV death, first HF hospitalization, and all-cause death, irrespective of both AF status and AF subtype (i.e. paroxysmal, persistent, and permanent).

## Introduction

Cardiac resynchronization therapy (CRT) represents a cornerstone in the treatment of patients with heart failure with reduced ejection fraction (HFrEF) on sinus rhythm and with prolonged QRS duration.^1^ The therapeutic response to CRT depends on the effective delivery of biventricular pacing, which atrial fibrillation (AF) may compromise through spontaneous, fusion, or pseudo-fusion beats.^2^ Evidence on the effectiveness of CRT in patients with AF remains inconclusive, as pivotal randomized controlled trials either included only a small proportion of patients with a history of AF or excluded those with concomitant AF at the time of randomization.^1,3–9^ A recent patient-level meta-analysis of randomized clinical trials found no prognostic benefit of CRT in patients with a history of AF. However, this analysis was markedly underpowered due to the limited number of patients with AF included.^10^ Similarly, observational evidence provided limited support for CRT use in this subset of patients, although most available studies are relatively dated.^11^

Therefore, it remains unclear whether advances in pharmacological and interventional management of AF, alongside the development of more effective CRT programming algorithms, might have improved the effectiveness of CRT in this subset of patients.

The aim of the current study was to assess the association between AF and CRT implantation, and whether AF modifies the associations between patients’ characteristics and CRT use, as well as between CRT and clinical outcomes, in a nationwide real-world cohort of patients with HFrEF meeting a class I or IIa indication for CRT according to current European HF guidelines.^1^

## Methods

Standardized reporting of the study plan is provided online (EU PAS number: 1000000699; https://catalogues.ema.europa.eu/catalogue-rwd-studies).

### Data sources

The study population was derived from the Swedish Heart Failure Registry (SwedeHF). The registry has been previously described in detail.^12^ In brief, it is an ongoing voluntary healthcare quality registry established in 2000 and nationally implemented in 2003. Although written consent is not mandatory, patients are informed about their inclusion in the national quality registry and can opt out. Most Swedish hospitals (69 out of 76) enrol patients from adult inpatient and outpatient settings, and, to a minor extent, also from primary care centres. It collects around 100 variables encompassing demographics, comorbidities, clinical parameters, biomarkers, treatments, and healthcare organization (http://www.swedehf.se). Until April 2017, the inclusion criterion was heart failure (HF) as judged by the treating clinician; thereafter, patients were included based on specific International Classification of Diseases-10 diagnostic codes for HF: I50.0, I50.1, I50.9, I42.0, I42.6, I42.7, I25.5, I11.0, I13.0, and I13.2.

For the current analysis, SwedeHF was linked by using the Swedish personal identity number, which all residents of Sweden have, to the following national registries.

The Swedish Implantable Cardioverter Defibrillator and Pacemaker (ICD/PM) Registry (http://www.pacemakerregistret.se) records both the date of CRT implantation and the type of device implanted [CRT-pacemaker(P)/CRT-defibrillator(D)], with more than 40 contributing centres covering > 95–98% of the total implantations in Sweden.

The National Patient Register reports additional data on comorbidities and HF hospitalization as outcome; the Cause of Death Register captures the date and cause of death. The National Prescribed Drug Register provides data about dispensed prescribed medications. The Longitudinal Integrated Database for health insurance and labour market studies (LISA) allows for obtaining socioeconomic data.^12^ A full description of our data sources is provided in the Supplementary material online, *Table S1*.

The definition of AF includes either a history of AF from the National Patient Register or evidence of AF rhythm on the electrocardiogram at the time of registration in SwedeHF.

### Study population

Patients registered in the SwedeHF between 1 January 2014 and 31 August 2022 with an ejection fraction (EF) < 40% and a HF duration >6 months (as a surrogate of optimal medical therapy) were considered for inclusion. We further selected patients who: a) received a CRT within 1 year before or 30 days after registration in SwedeHF, or b) had QRS duration ≥ 150 ms (i.e. meeting a class I or IIa indications for CRT according to the current European HF guidelines) but did not undergo implantation before or until 30 days after index date (control group).^1^ We excluded the patients who underwent His bundle ablation prior to or at the time of the index date. Index date was defined as the date of CRT implantation for the CRT population and the date of registration in SwedeHF for the controls (i.e. date of clinical visit for outpatients and date of discharge for inpatients). If multiple registrations in SwedeHF were available, the one closest to the date of CRT implantation was selected for patients with CRT, and the first available was used for the control group (see Supplementary material online, *Figure S1*).

### Outcomes

The primary endpoint was the composite of time to cardiovascular (CV) death or first HF hospitalization. Secondary endpoints were time to first HF hospitalization, CV death, all-cause death, and the total number of HF hospitalizations. A negative control (falsification) analysis was also run to test the association between CRT use and the composite outcome of time to first hospitalization for trauma or genitourinary tract infection. As such an association would be pathophysiologically implausible, its presence would indicate residual confounding. Follow-up data were censored on 31 December 2023, at emigration from Sweden or at death from other causes than the event. In the cross-over sensitivity analysis, follow-up was censored on 31 August 2022, as data from the ICD/PM Registry were only available up to that date.

### Statistical analysis

Categorical variables were reported as numbers (percentages) and compared using a χ^2^ test, whereas continuous variables were reported as medians (1st quartile- 3rd quartile) and compared by the Kruskal–Wallis test according to CRT use and history of or concomitant AF. Missing data for covariates included in the multivariable models were handled by multiple imputations by chained equations, with 10 imputed datasets generated. The percentage of missing data is reported in *Table 1*.

**Table 1:** Baseline characteristics according to CRT implantation and AF

Variables	No-CRT^ab^	CRT ^ab^	*P* value	No-AF	AF	*P* value	Missing %
**N (%)**	2657 (75.3%)	873 (24.7%)	—	1708 (48.4)	1822 (51.6)	—	0
**AF (%)** ^ab^	1355 (51)	467 (53.5)	0.200	—	**Type of AF** Paroxysmal 372 (20.4^c^) Persistent 137 (7.5^c^) Permanent 669 (36.7^c^)	—	35.3^c^

—	—	—	—	No-CRT	CRT	*P* value	No-CRT	CRT	*P* value	—	—
**N (%)**	—	—	—	1302 (76.2)	406 (23.8)	—	1355 (74.4)	467 (25.6)	—	0.200	0
**CRT type**	—		—	—		—	—		—	**<0.001**	0
CRT-D		591 (67.7)			311 (76.6)			280 (60)			
CRT-P		282 (32.3)			95 (23.4)			187 (40)			
**Calendar year** ^ab^			**0**.**033**			0.600			**0**.**011**	**0.002**	0
2014–2017	1319 (49.6)	397 (45.5)		603 (46.3)	182 (44.8)		716 (52.8)	215 (46)			
2018–2022	1338 (50.4)	476 (54.5)		699 (53.7)	224 (55.2)		639 (47.2)	252 (54)			
**Follow-up in nurse-led HF unit** ^ab^	2080 (81.6)	712 (84.4)	0.069	1051 (83.4)	339 (86.3)	0.169	1029 (79.9)	373 (82.7)	0.193	**0.009**	3.8
**Follow-up location** ^ab^			**<0.001**			**<0.001**			**<0.001**	**0.018**	2.3
— *Hospital*	2024	818 (94.7)		1033 (80.8)	383 (95)		991 (75.8)	435 (94.4)			
— *Primary care*	(78.3)	36 (4.2)		212 (16.6)	16 (4)		280 (21.4)	29 (4.3)			
— *Other*	492 (19) 69 (2.7)	10 (1.2)		33 (2.6)	4 (1)		36 (2.8)	6 (1.3)			
**Sex** ^ab^			0.631			0.745			0.567	**<0.001**	0
— *Male*	2054 (77.3)	668 (76.6)		944 (72.5)	291 (71.7)		1110 (81.9)	377 (80.7)			
**Duration of HF** (days)	1934 (679–3766)	1912 (517–3885)	0.219	1555.5 (502–3543)	1417.5 (343–3680)	0.095	2196 (909–3985)	2303 (760–4085)	0.713	**<0.001**	0
**Duration of HF** **>** **median** ^ab^	1330 (50.1)	435 (49.8)	0.907	582 (44.7)	174 (42.9)	0.514	748 (55.2)	261 (55.9)	0.797	**<0.001**	0
**EF** ^ab^			**<0.001**			0.056			**0**.**002**	0.145	0
<30%	1324 (49.8)	496 (56.8)		638 (49)	221 (54.4)		686 (50.6)	275 (58.9)			
30–39%	1333 (50.2)	377 (43.2)		664 (51)	185 (45.6)		669 (49.4)	192 (41.1)			
**QRS duration** (ms)	162 (155–172)	150 (134–166)	**<0.001**	162 (155–172)	149.5 (135–166)	**<0.001**	162 (154–173)	150 (132–166)	**<0.001**	0.444	4.9
**QRS duration** **≥** **150** (ms) ^a^	2657 (100)	354 (50.6)	**<0.001**	1302 (100)	161 (50)	**<0.001**	1355 (100)	193 (51.1)	**<0.001**	0.468	4.9
**LBBB** ^ab^	1761 (72.2)	239 (68.5)	0.149	952 (77.7)	120 (76)	0.617	809 (66.7)	119 (62.3)	0.240	**< 0.001**	21
**SBP** (mmHg)	120 (110–135)	117 (105–130)	**<0.001**	122 (110–136)	120 (108–130)	**<0.001**	120 (108–132)	115 (104–130)	**<0.001**	**<0.001**	3.2
**SBP** (mmHg) **>** 110 ^ab^	1790 (69.2)	509 (61.3)	**<0.001**	931 (73.5)	256 (66.3)	**0**.**006**	859 (65.1)	253 (56.9)	**0**.**002**	**<0.001**	3.2
**DBP** (mmHg)	70 (63–80)	70 (62–80)	0.952	70 (64–80)	70 (63–80)	0.770	70 (62–80)	70 (62–80)	0.686	**0.013**	3.1
**MBP** (mmHg)	88.3 (80–96.7)	86.7 (78.3–94)	**0**.**005**	89.3 (81–96.7)	87.7 (78.7–95)	**0**.**023**	86.7 (78.7–95)	86 (78.3–93.3)	0.098	**<0.001**	3
**MBP** (mmHg) > 90 ^ab^	1042 (40.2)	306 (36.9)	0.086	544 (42.9)	154 (39.9)	0.295	498 (37.6)	152 (34.2)	0.198	**0.001**	3
**HR** (beats/min)	70 (60–79)	70 (62–78)	0.313	67 (60–76)	67 (61–76)	0.286	71 (62–81)	70 (64–79)	0.786	**<0.001**	3.2
**HR** (beats/min) > 70 ^ab^	1187 (45.4)	352 (43.9)	0.439	491 (38.4)	142 (38.9)	0.859	696 (52.2)	210 (48.1)	0.131	**<0.001**	3.2
**eGFR** (mL/min/1.73m^2^)	61.9 (46.3–81.6)	64.5 (49.3–83)	**0**.**031**	68.5 (49.7–87.1)	72.4 (53.1–89.1)	**0**.**018**	57.1 (43.5–75.5)	58.8 (45.9–74.3)	0.256	**<0.001**	2.6
**eGFR** (mL/min/1.73m^2^) ≥ 60 ^ab^	1374 (52.6)	468 (56.7)	**0**.**043**	777 (61.2)	260 (67.5)	**0**.**024**	597 (44.5)	208 (47.2)	0.333	**<0.001**	2.6
**NT-pro BNP** (pg/mL)	2163 (931–5368)	1998 (782–4630)	**0**.**042**	1430 (605–4045)	1244 (483–3000)	**0**.**026**	2927 (1450–6615)	2870 (1354–5703)	0.192	**<0.001**	23
**NT-pro BNP** (pg/mL) > median ^ab^	1046 (50.6)	312 (48.2)	0.300	406 (39.4)	102 (34.2)	0.107	640 (61.7)	210 (60.2)	0.622	**<0.001**	23
**NYHA class** ^ab^			0.920			0.942			0.828	**<0.001**	19.9
NYHA I-II	1190 (55.9)	389 (55.7)		681 (63.2)	209 (62.9)		509 (48.5)	180 (49.2)			
NYHA III-IV	937 (44.1)	309 (44.3)		397 (36.8)	123 (37.1)		540 (51.5)	186 (50.8)			
**Prior PM/ICD implantation**	—	123 (14.1)	—	—	41 (10.1)	—	—	82 (17.6)	—	**0.002**	0
**CRT implantation after index date**	697 (26.2)	—	—	388 (29.8)	—	—	309 (22.8)	—	—	**<0.001**	0
**Age**	76 (69–83)	73 (66–78)	**<0.001**	74 (66–81)	71 (63–77)	**<0.001**	78 (72–84)	75 (69–80)	**<0.001**	**<0.001**	0
**Age** **≥** 75 years ^ab^	1514 (57)	394 (45.1)	**<0.001**	630 (48.4)	150 (37)	**<0.001**	884 (65.2)	244 (52.3)	**<0.001**	**<0.001**	0
**Children** ^ab^	2231 (83.9)	735 (84.2)	0.875	1085 (83.3)	336 (82.7)	0.787	1146 (84.6)	399 (85.4)	0.654	0.195	0
**Family situation** ^ab^ *Living alone*	1205 (45.5)	358 (41)	**0**.**025**	612 (47)	173 (42.6)	0.121	593 (43.8)	185 (39.6)	0.118	0.051	0
**Education** ^ab^			**0**.**003**			0.665			**<0.001**	0.197	1.7
— *University*	470 (17.9)	185 (21.7)		242 (18.9)	78 (19.6)		228 (17.1)	107 (23.6)			
— *Secondary school*	1081 (41.3)	372 (43.6)		549 (42.8)	178 (44.6)		532 (39.9)	194 (42.8)			
— *Compulsory school*	1066 (40.7)	295 (34.6)		492 (38.4)	143 (35.8)		574 (43)	152 (33.6)			
**Income**	1690 (1406–2254)	1800 (1474–2553)	**<0.001**	1700 (1397–2377)	1818 (1466–2611)	**0**.**006**	1679 (1409–2131)	1789 (1477–2501)	**0**.**002**	0.107	0
**Income** > median ^ab^	1280 (48.2)	485 (55.6)	**<0.001**	640 (49.2)	229 (56.4)	**0**.**011**	640 (47.2)	256 (54.8)	**0**.**005**	0.312	0
**Hypertension** ^ab^	1734 (65.3)	549 (62.9)	0.203	794 (61)	236 (58)	0.305	940 (69.4)	313 (67)	0.345	**<0.001**	0
**Diabetes** ^ab^	822 (30.9)	268 (30.7)	0.895	381 (29.3)	116 (28.6)	0.789	441 (32.6)	152 (32.6)	0.999	**0.027**	0
**Ischaemic heart disease** ^a b^	1703 (64.1)	555 (63.6)	0.781	791 (60.8)	244 (60.1)	0.814	912 (67.3)	311 (66.6)	0.778	**<0.001**	0
**PCI**	740 (27.9)	259 (29.7)	0.301	368 (28.3)	126 (31)	0.282	372 (27.5)	133 (28.5)	0.669	0.427	0
**CABG**	1015 (38.2)	331 (37.9)	0.880	448 (34.4)	136 (33.5)	0.735	567 (41.9)	195 (41.8)	0.973	**<0.001**	0
**PAD** ^a b^	273 (10.3)	82 (9.4)	0.452	116 (8.9)	31 (7.6)	0.424	157 (11.6)	51 (10.9)	0.696	**0.006**	0
**Stroke/TI** ^ab^	394 (14.8)	114 (13.1)	0.196	152 (11.7)	34 (8.4)	0.062	242 (17.9)	80 (17.1)	0.722	**<0.001**	0
**Valvular disease** ^a b^	641 (24.1)	184 (21.1)	0.065	255 (19.6)	50 (12.3)	**0**.**001**	386 (28.5)	134 (28.7)	0.932	**<0.001**	0
**COPD** ^a b^	327 (12.3)	109 (12.5)	0.889	157 (12.1)	55 (13.6)	0.427	170 (12.6)	54 (11.7)	0.577	0.915	0
**Liver disease** ^a b^	61 (2.3)	18 (2.1)	0.685	28 (2.2)	9 (2.2)	0.936	33 (2.4)	9 (1.9)	0.528	0.780	0
**Dementia** ^a b^	32 (1.2)	2 (0.2)	**0**.**010**	13 (1)	0 (0)	**0**.**043**	19 (1.4)	2 (0.4)	0.089	0.234	0
**Malignant cancer within 3 years** ^a b^	369 (13.9)	82 (9.4)	**0**.**001**	140 (10.8)	38 (9.4)	0.422	229 (16.9)	44 (9.4)	**<0.001**	**<0.001**	0
**Muscolo-skeletal/connective tissue diseases within 3 years** ^a b^	830 (31.2)	285 (32.7)	0.438	388 (29.8)	128 (31.5)	0.508	442 (32.6)	157 (33.6)	0.692	0.089	0
**Bleeding** ^a b^	527 (19.8)	128 (14.7)	**0**.**001**	203 (15.6)	48 (11.8)	0.061	324 (23.9)	80 (17.1)	**0**.**002**	**<0.001**	0
**ICD**	311 (11.7)	—	**—**	119 (9)	—	—	192 (14.2)	—	**—**	**<0.001**	0
**PM**	319 (12)	—	**—**	121 (9.3)	—	—	198 (14.6)	—	—	**<0.001**	0
**BB** ^ab^	2383 (89.7)	794 (90.6)	0.280	1166 (89.6)	373 (91.9)	0.172	1217 (89.8)	421 (90.2)	0.836	0.840	0
**RASi/ARNI** ^ab^	2412 (90.8)	818 (93.7)	**0**.**007**	1207 (92.7)	381 (93.8)	0.433	1205 (88.9)	437 (93.6)	**0**.**004**	**0.002**	0
**MRA** ^ab^	1569 (59.1)	619 (70.9)	**<0.001**	801 (61.5)	302 (74.4)	**<0.001**	768 (56.7)	317 (67.9)	**<0.001**	**0.002**	0
**SGLT2i** ^ab^	239 (9)	100 (11.5)	**0**.**032**	125 (9.6)	42 (10.3)	0.659	114 (8.4)	58 (12.4)	**0**.**011**	0.734	0
**Loop diuretic** ^ab^	1814 (68.3)	545 (62.4)	**0**.**001**	787 (60.5)	218 (53.7)	**0**.**016**	1027 (75.8)	327 (70)	**0**.**014**	**<0.001**	0
**Nitrates** ^ab^	622 (23.4)	177 (20.3)	0.055	309 (23.7)	78 (19.2)	0.057	313 (23.1)	99 (21.2)	0.397	0.974	0
**Anti-platelet** ^ab^	1106 (41.6)	332 (38)	0.061	810 (62.2)	240 (59.1)	0.263	296 (21.9)	92 (19.7)	0.329	**<0.001**	0
**Anti-coagulant** ^ab^	1262 (47.5)	458 (52.5)	**0**.**011**	178 (13.7)	60 (14.8)	0.574	1084 (80)	398 (85.2)	**0**.**012**	**<0.001**	0
**Lipid lowering** ^ab^	1563 (58.8)	539 (61.7)	0.128	794 (61)	246 (61)	0.888	769 (56.8)	293 (62.7)	**0**.**024**	0.115	0
**CCB** ^ab^	327 (12.3)	74 (8.5)	**0**.**002**	163 (12.5)	33 (8.1)	**0**.**015**	164 (12.1)	41 (8.8)	0.05	0.834	0
**Insulin** ^ab^	370 (13.9)	121 (13.9)	0.961	184 (14.1)	51 (12.6)	0.423	186 (13.7)	70 (15)	0.498	0.802	0
**Oral anti-diabetic** ^ab^	630 (23.7)	234 (26.8)	0.065	319 (24.5)	101 (24.8)	0.878	311 (23)	133 (28.5)	**0**.**016**	0.879	0
**Digoxin** ^ab^	259 (9.8)	114 (13.1)	**0**.**006**	23 (1.7)	8 (2)	0.788	236 (17.4)	106 (22.7)	**0**.**012**	**<0.001**	0
**Anti-arrhythmic** ^ab^	171 (6.4)	106 (12.1)	**<0.001**	41 (3.2)	20 (4.9)	0.092	130 (9.6)	86 (18.4)	**<0.001**	**<0.001**	0
**Pulmonary vein isolation/electrical cardioversion**	—	—	**—**	—	—	—	217 (16%)	121 (25.9%)	**<0.001**	**—**	0

*P* values < 0.05 are shown in bold.

AF, atrial fibrillation; ARNI, angiotensin receptor/neprilysin inhibitor; BB, beta-blocker; CABG, coronary artery bypass grafting; CCB, calcium channel blocker; COPD, chronic obstructive pulmonary disease; CRT, cardiac resynchronization therapy; DBP, diastolic blood pressure; EF, ejection fraction; eGFR, estimated glomerular filtration rate; HF, heart failure; HR, heart rate; ICD, implantable cardioverter-defibrillator; LBBB, left bundle branch block; MBP, mean blood pressure; MRA, mineralocorticoid receptor antagonist; *N*, number; NT-pro BNP, N-terminal pro b-type natriuretic peptide; NYHA, New York Heart Association; PAD, peripheral artery disease; PCI, percutaneous coronary intervention; PM, pace-maker; RASi, renin–angiotensin system inhibitor; SBP, systolic blood pressure; SGLT2i, Sodium-Glucose Cotransporter 2 inhibitors; TIA, Transient ischaemic attack.

^a^Included in multiple imputation and multivariable Cox regression models.

^b^Included in logistic regression model and propensity score calculation.

^c^Here percentages are calculated considering only patients with AF.

Patients’ characteristics associated with CRT use were assessed by using multivariable logistic regression models, with an interaction term between AF and each covariate. The results were expressed as an odds ratio (OR) with a 95% confidence interval (CI).

We investigated the crude annual (index year) percentage of CRT implantation in our cohort, which was then stratified by the presence or history of AF. The association between CRT use and calendar year was also assessed by a multivariable logistic regression model, adjusting for baseline patient characteristics (linear trend test).

The propensity score for CRT use was separately calculated in each imputed dataset by a logistic regression model and then averaged across the 10 imputed datasets, using the ‘across’ approach.^13^ Overlap weighting (OW) was then applied, using the average treatment effect in the overlap population as the estimand, to ensure balance in baseline characteristics between the weighted comparison groups.^14^ Baseline covariate balance between CRT users and non-users was assessed by using standardized mean differences, with values <10% indicating acceptable balance. The variables included in the imputation and multiple regression models are those marked with (^χ^*) in *Table 1*, together with the primary outcome, which was also included in the multiple imputation model. To investigate the association between CRT use and time-to-event outcomes, univariable, weighted with OW, and multivariable (adjusted for variables in *Table 1*) Cox proportional hazards regression models with robust standard errors were fitted. The results were expressed as a hazard ratio (HR) with 95% CI. The association between CRT use and the total number of HF hospitalizations was assessed by using univariable, weighted with OW, and multivariable (adjusted for variables in *Table 1*) negative binomial regression models, using the logarithm of follow-up time as an offset. The results were expressed as incidence rate ratios (IRR) with 95% CI. An interaction term between CRT use and AF was included in the models to assess whether the association of CRT with clinical outcomes varied by AF status. Survivor functions in patients with CRT vs. control were estimated using the non-parametric Kaplan-Meier method in the overall population, and after applying OW in the weighted population.^15^ The proportional hazards assumption was verified by assessing Schoenfeld residuals and was met. All statistical analyses were performed using Stata version 18.1 (Stata Corp., College Station, TX). A *P*-value <0.05 (two sided) was considered as statistically significant.

### Subgroup analyses

Two pre-specified subgroup analyses were conducted: 1) the exposure variable was categorized into three levels (i.e. no-CRT, CRT-P, and CRT-D), with no-CRT serving as the reference category, to independently assess the association of CRT-P and CRT-D with clinical outcomes as compared with no-CRT. For this analysis, adjustment for potential confounding was performed using only a multivariable model, as the propensity score was specifically calculated for CRT use, defined as a binary variable (CRT vs. no-CRT). 2) AF was categorized into four levels (i.e. no-AF, paroxysmal AF, persistent AF, and permanent AF) to assess whether the association between CRT use and clinical outcomes varied according to AF subtypes. Patients with AF of unknown type were excluded from this analysis.

### Cross-over sensitivity analysis

The association of CRT use with clinical outcomes was further assessed after excluding patients in the control group who underwent CRT implantation during follow-up.

### Contemporary-cohort sensitivity analysis (2017–2022)

To assess the association between CRT use and clinical outcomes in a setting reflecting the most recent clinical practice, we conducted a sensitivity analysis restricted to patients registered in 2017 or later, corresponding to the period following the publication of the 2016 European HF guidelines.^16^

### Sensitivity analyses using alternative definitions of AF

The consistency of the results was further assessed considering two alternative definitions of AF: 1) defined only as evidence of AF rhythm on the electrocardiogram at the time of registration in SwedeHF, and 2) as either AF on the electrocardiogram or as a history of AF from the National Patient Register within one year of the index date.

### Sensitivity analysis excluding patients who underwent his bundle ablation during follow-up

Since some patients may have undergone His ablation after CRT implantation to ensure effective biventricular pacing, we further assessed the robustness of our findings by excluding these patients.

### Sensitivity analysis excluding patients with AF who underwent pulmonary vein isolation during follow-up

Since some patients with AF may have undergone pulmonary vein isolation during follow-up to reduce AF burden and promote more effective biventricular pacing, we assessed the consistency of the results by excluding these patients.

In the subgroup and sensitivity analyses, propensity scores and the corresponding OW were recalculated after excluding patients.

## Results

**Figure 1:**
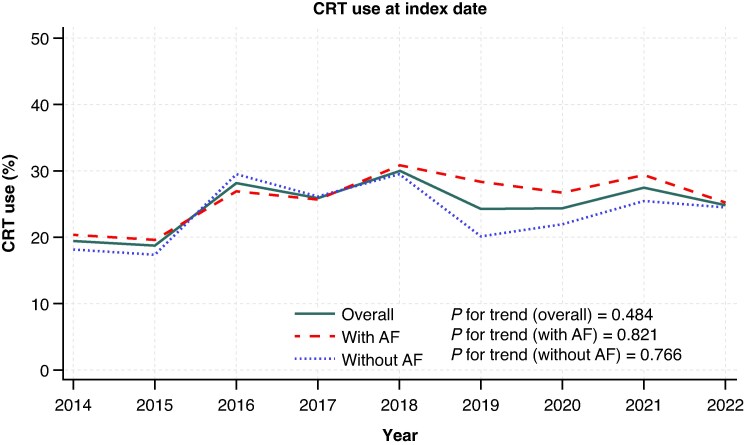
CRT use at index date. The figure shows the annual crude rate of CRT use at the index year among eligible patients, also stratified according to AF status. AF, atrial fibrillation; CRT, cardiac resynchronization therapy.

Between 1 January 2014 and 31 August 2022, a total of 3530 patients with HFrEF and an indication for CRT met the selection criteria for inclusion in the current study [median age of 75 years (68 years–82 years), and 2722 (77.1%) males]. In the CRT group, 23.5% of patients (377 of 1607) were previously excluded because they had undergone His-bundle ablation prior to or at the time of device implantation (see Supplementary material online, *Figure S1*). Of the total study population, 873 patients (24.7%) received CRT, while the remaining 2657 (75.3%) did not, and were included in the control group (*Table 1*). Within the overall cohort, 1822 patients (51.6%) had a history of or concomitant AF, of whom 467 (25.6%) received a CRT; the remaining 1708 patients (48.4%) had no history of, or concomitant AF, with 406 (23.8%) receiving CRT. The. Over time, there was no statistically significant increase in CRT use at inclusion in the study, either in the overall population (19.3% in 2014, 24.8% in 2022, *P* for linear trend = 0.484) or when stratified by AF (AF patients: 20.3% in 2014, 25.2% in 2022, *P* for linear trend = 0.821; no-AF patients: 18.1% in 2014, 24.4% in 2022, *P* for linear trend = 0.766) (*Figure 1*).

### Baseline characteristics according to CRT implantation and AF status

Baseline characteristics of patients who received vs. did not receive a CRT, both overall and according to AF status, are presented in *Table 1*. A significantly greater proportion of CRT-P type was observed in patients with AF as compared with those without AF. Among patients who received CRT, those with AF had a higher prevalence of prior pacemaker or implantable cardioverter defibrillator implantations as compared with those without AF (17.6% vs. 10.1%, respectively; *P* = 0.002) (*Table 1*). Among patients with AF, 16% of the no-CRT group and 25.9% of the CRT group had undergone pulmonary vein isolation or electrical cardioversion (*P* < 0.001) (*Table 1*). Patients who received CRT were younger, more frequently managed in the hospital setting and more often had EF values <30%, regardless of AF status. A history of dementia, cancer, and bleeding was less common among patients who received CRT as compared with those who did not, irrespective of AF status.

### Independent predictors of CRT use

A history of or concomitant AF was not independently associated with CRT use.

**Figure 2:**
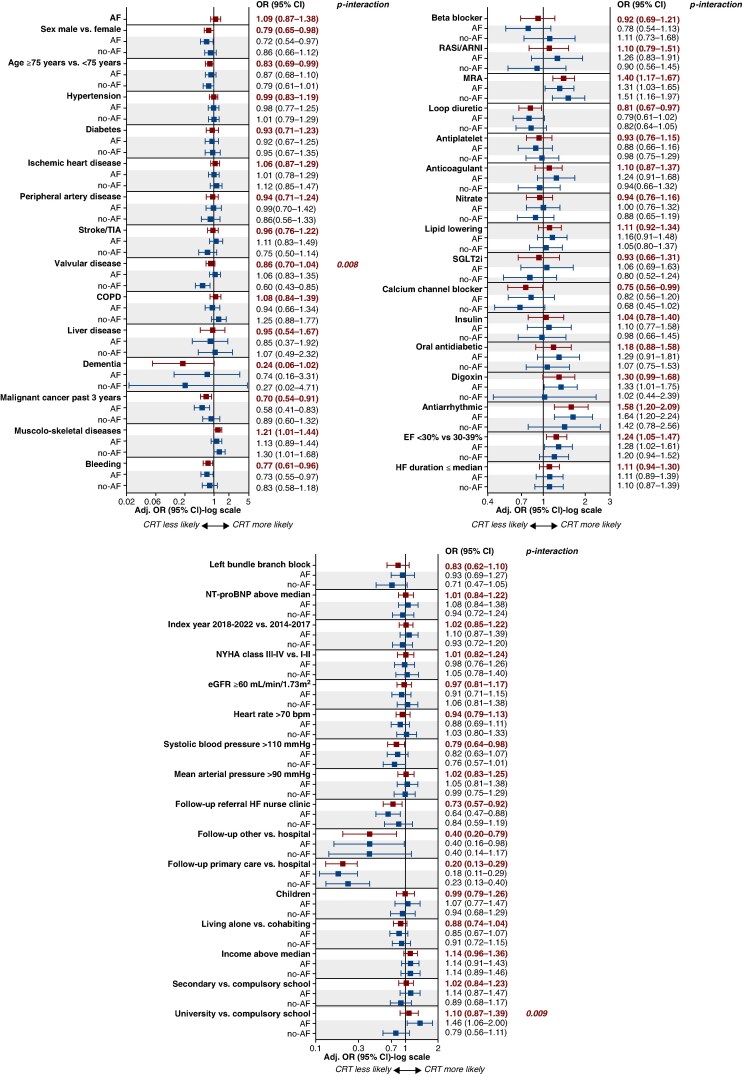
Predictors of CRT use. Adjusted OR and 95% CI are presented for the association between baseline patient characteristics and CRT use in the multivariable logistic regression analysis. The figure includes results for the overall population and by AF status (only interaction *P* values < 0.05 are shown). Adj, adjusted; AF, atrial fibrillation; ARNI, angiotensin receptor/neprilysin inhibitor; CI, confidence interval; COPD, chronic obstructive pulmonary disease; CRT, cardiac resynchronization therapy; EF, ejection fraction; eGFR, estimated glomerular filtration rate; HF, heart failure; MRA, mineralocorticoid receptor antagonist; NYHA, New York Heart Association classification; NT-pro BNP, N-terminal pro b-type natriuretic peptide; OR, odds ratio; RASi, renin–angiotensin system inhibitor; SGLT2i, Sodium-Glucose Cotransporter 2 inhibitors; TIA, transient ischaemic attack.

Independent predictors of CRT implantation included lower EF, musculoskeletal disease, use of mineralocorticoid receptor antagonists (MRA), and use of anti-arrhythmic drugs (*Figure 2*). Conversely, follow-up in a nurse-led HF unit, follow-up in primary care or other facilities, as compared with hospital, male sex, history of cancer, bleeding, use of loop diuretics or calcium channel blockers, systolic blood pressure > 110 mmHg, and age ≥ 75 years were independently associated with a lower likelihood of CRT implantation (*Figure 2*).

Significant interactions with AF were only found for valvular disease and educational level. Specifically, among patients without AF, those without valvular disease were more likely to receive CRT as compared with those with valvular disease. Among patients with AF, those with a university-level education were more likely to receive CRT as compared with those with compulsory education (*P* for interaction = 0.008 and 0.009, respectively) (*Figure 2*).

### Outcome analysis

Over a median follow-up of 3.3 years (1.7 years–5.4 years), 913 (53.5%) patients in the no-AF group and 1194 (65.5%) patients in the AF group experienced the composite outcome of first HF hospitalization or CV death. Seven hundred and four (41.2%) patients in the no-AF group and 1047 (57.5%) patients in the AF group died from any cause. After weighing, all baseline covariates achieved a standard mean difference <10% (see Supplementary material online, *Table S2* and *Figure S2*).

### Time to CV death/first HF hospitalization

**Figure 3:**
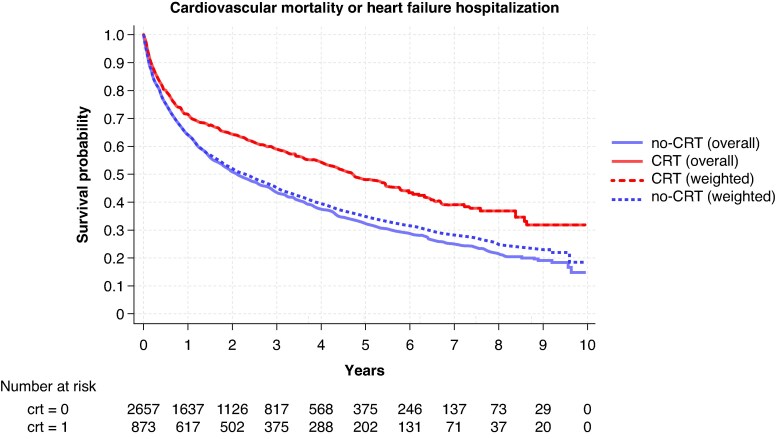
Kaplan–Meier survival estimates for the primary outcome of time to cardiovascular death or first heart failure hospitalization in the overall and weighted populations. CRT, cardiac resynchronization therapy.

**Figure 4:**
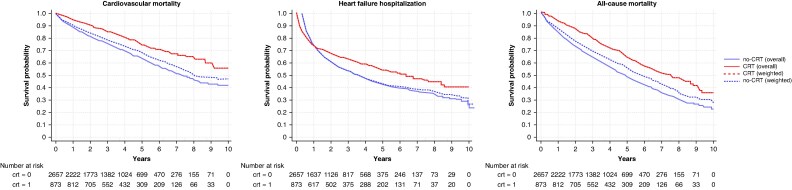
Kaplan–Meier survival estimates for the time to cardiovascular death, time to first heart failure hospitalization, and time to all-cause death in the overall and weighted populations. CRT, cardiac resynchronization therapy.

In the OW Cox regression analysis, the implantation of CRT was independently associated with a lower risk of the composite of CV death or first HF hospitalization (HR: 0.71; 95% CI: 0.64–0.79), both in patients with AF (HR: 0.73; 95% CI: 0.63–0.84) and in patients without AF (HR: 0.68; 95% CI: 0.57–0.81) (see Supplementary material online, *Table S3*, *Figure 3*). When the components of the primary outcome were analysed separately, CRT use was independently associated with a lower risk of CV death (AF: HR 0.68, 95% CI 0.56–0.83; no-AF: HR 0.71, 95% CI 0.55–0.92) and first HF hospitalization (AF: HR 0.78, 95% CI 0.67–0.91; no-AF: HR 0.70, 95% CI 0.58–0.84), with no significant interaction according to AF status (see Supplementary material online, *Table S3*, *Figure 4*). All the associations between CRT use and the aforementioned outcomes were consistent when a multivariable model was rather performed (see Supplementary material online, *Table S4*).

### Time to all-cause death

In the Cox regression model weighted by OW, use of CRT was associated with a lower risk of all-cause death (HR: 0.72; 95% CI: 0.64–0.81), which was consistent in patients with (HR: 0.73; 95% CI: 0.63–0.86) and in those without AF (HR: 0.70; 95% CI: 0.57–0.85) (see Supplementary material online, *Table S3*, *Figure 4*). Consistent results were shown when a multivariable Cox regression analysis was applied (see Supplementary material online, *Table S4*).

### Total number of HF hospitalizations

In the multivariable and weighted negative binomial regression analyses, CRT use was not associated with the total HF hospitalizations rate, regardless of AF status (see Supplementary material online, *Tables S5*, *S6*).

### Subgroup analyses

#### CRT subtype

The association between CRT use and a lower risk of the primary endpoint, the individual components, and of all-cause death was consistent when CRT-P and CRT-D were analysed separately, as compared with no-CRT (see Supplementary material online, *Table S7*), with no significant interaction by AF status. No significant association was observed between each CRT subtype and the number of HF hospitalizations, as compared with patients without CRT (see Supplementary material online, *Table S6*).

#### AF subtypes

Data on AF subtype were missing for 35% of those with AF, who were consequently excluded from this subgroup analysis, which therefore included a total of 2886 patients—773 (26.8%) with CRT and 2113 (73.2%) without CRT (*Table 1*). The association between CRT use and lower risk of all-cause death, CV death, and first HF hospitalization was consistent across all AF subtypes (see Supplementary material online, *Tables S8* and *S9*). In the multivariable negative binomial regression model, AF subtype significantly influenced the association between CRT use and the number of HF hospitalizations (*P* for interaction = 0.019): among patients with paroxysmal AF, CRT use was associated with a tendency towards higher rates of total HF hospitalizations (IRR 1.52; 95% CI: 0.99–2.34) (see Supplementary material online, *Table S10*).

### Cross-over sensitivity analysis

During follow-up, 697 patients in the control group (26.2%) underwent CRT implantation (median time from index date to implantation: 230 days; 96 days–489 days) and were excluded from the cross-over sensitivity analysis (see Supplementary material online, *Table S11*). The association between CRT and a lower risk of CV death and all-cause death was consistent in both the multivariable and OW Cox regression models, regardless of AF status (see Supplementary material online, *Tables S12*, *S13*). Conversely, no statistically significant association was observed between CRT and either the risk of first HF hospitalization or the number of HF hospitalizations, with no significant interaction by AF status (see Supplementary material online, *Tables S5*, *S6*, *S12*, and *S13*).

### Contemporary-cohort sensitivity analysis

A total of 2267 patients with a registration year from 2017 onwards were included, of whom 593 (26.2%) received CRT and 1674 (73.8%) did not. The association between CRT and a lower risk of the primary outcome, first HF hospitalization, CV death, and all-cause death was consistent at the multivariable and OW Cox regression models, with no significant interaction by AF status (see Supplementary material online, *Tables S14*, *S15*). CRT use was associated with a lower rate of total HF hospitalizations in patients without AF in the multivariable model (no-AF: IRR 0.68; 95% CI 0.51–0.91; *P* for interaction: 0.017), but not in the OW model (see Supplementary material online, *Table S16*).

### Sensitivity analyses using alternative definitions of AF

An electrocardiographic rhythm diagnosis was available for 3428 patients (97.1%) at the time of registration in SwedeHF, of whom 893 (26.1%) had AF (16% in the CRT group vs. 29% in the no-CRT group). CRT use was associated with a lower risk of the primary outcome, first HF hospitalization, CV death, and all-cause death, independent of the presence of AF at the electrocardiogram. 1654 patients (47%) had AF (50% in the CRT group vs. 46% in the no-CRT group), defined by electrocardiographic evidence at the time of registration in SwedeHF or by a comorbidity diagnosis from the National Patient Registry within the year prior to the index date. The association between CRT and a lower risk of the primary outcome, first HF hospitalization, CV death, and all-cause death was consistent regardless of the latter definition of AF. No association was found between CRT use and the total number of HF hospitalizations, regardless of either definition of AF (see Supplementary material online, *Tables S17* and *S18*).

### Sensitivity analysis excluding patients who underwent his bundle ablation during follow-up

246 patients (6.9%) underwent His bundle ablation during follow-up [78 (8.9%) in the CRT group and 168 (6.3%) in the no-CRT group] and were excluded from the current sensitivity analysis. The association between CRT and a lower risk of the primary outcome, first HF hospitalization, CV death, and all-cause death was consistent at the multivariable and OW Cox regression models, with no significant interaction by AF status. No statistically significant association was observed between CRT and the number of HF hospitalizations, with no significant interaction by AF status (see Supplementary material online, *Table S19*).

### Sensitivity analysis excluding patients with AF who underwent pulmonary vein isolation during follow-up

During follow-up, 66 patients (3.6% of those with AF) underwent pulmonary vein isolation and were excluded from the current sensitivity analysis. The association between CRT and a lower risk of the primary outcome, first HF hospitalization, CV death, and all-cause death was consistent at the multivariable and OW Cox regression models, with no significant interaction by AF status. No statistically significant association was observed between CRT and the number of HF hospitalizations, with no significant interaction by AF status (see Supplementary material online, *Table S20*).

### Negative control analysis

There was no significant association between CRT use and the composite risk of time to first hospitalization for trauma or genitourinary tract infection in patients with and without AF, in either the OW or multivariable Cox regression models, supporting the assumption of minimal residual confounding (see Supplementary material online, *Tables S21* and *S22*).

## Discussion

The main findings of the current study, which included 3530 patients with HFrEF and an indication for CRT between 2014 and 2022 from SwedeHF, were:

1. CRT use at index date remained low and stable over time, regardless of AF, ranging in the overall eligible population from 19% in 2014 to 25% in 2022, in patients with AF from 20% in 2014 to 25% in 2022, and in patients without AF from 18% in 2014 to 24% in 2022.
2. A history or concurrent presence of AF was not associated with the likelihood of receiving a CRT and, in most cases, did not act as a modifier of the association between other patient characteristics and likelihood of CRT implantation;
3. CRT use was associated with a lower risk of all-cause death, CV death, and first HF hospitalization, both in patients with and without AF, and regardless of AF subtype (*Graphical Abstract*).

### Temporal trends in CRT use

Patients included in the present study had a strong recommendation (class I or IIa, depending on the presence or absence of left bundle branch block and AF) for CRT implantation according to the latest HF European guidelines.^1^ During the study period, no linear trend was observed in the likelihood of CRT implantation irrespective of AF status, suggesting that therapeutic inertia has remained largely unchanged over time in both AF and non-AF patients. Part of the plateau in CRT implantation rates over time might be attributable to the increasing interest in the use of conduction system pacing as an alternative to conventional biventricular pacing.^17^ A comparable implantation rate was observed in a United States registry-based cohort study conducted between 2008 and 2014, encompassing 6013 patients who met consistent electrocardiographic criteria for CRT implantation.^18^ In contrast, a European registry, enrolling 1031 patients between 2013 and 2016, reported a slightly higher implantation rate (47%), which may be attributable to the predominant inclusion of CRT-implanting centres.^19^ In the current study, a higher proportion of patients with AF than those without received CRT as an upgrade from a pacemaker or defibrillator, likely reflecting a more severe and advanced disease. Upgrading to CRT in patients with intermittent or permanent right ventricular pacing, irrespective of baseline rhythm, is supported by a pre-specified analysis of the BUDAPEST trial, which demonstrated comparable efficacy between patients with AF and those in sinus rhythm.^20^ Notably, in our study, around a quarter of patients in the control group underwent CRT implantation during the follow-up period, with a median time of 230 days from the index date. At that date, a guideline-based indication for CRT was ascertained, and patients were likely to be already on optimal medical therapy, given the selection of a population with HF duration >6 months. These findings might suggest that therapeutic inertia can manifest either as a failure to implant or as a delay in implantation, with the latter also being associated with a higher risk of adverse CV events as compared with early CRT implantation.^21^ Therefore, our findings underscore the urgency of implementing measures to promote its broader and prompt adoption, regardless of AF status.

### Predictors of CRT use

In this study, we investigated patient characteristics significantly associated with the likelihood of CRT implantation and assessed whether the presence of AF modified their associations with the likelihood of CRT implantation. Identifying patient subgroups in which CRT remains underutilized could provide a foundation for developing targeted strategies to support its broader implementation. Notably, a history of, or concomitant AF, was not associated with less likely implantation of CRT, despite the less robust evidence supporting its efficacy in this population, as compared with patients in sinus rhythm.^1^ As inadequate rate control in AF, impairing the possibility of delivering biventricular pacing, has been the major obstacle for CRT in AF patients, our finding may hypothetically point to improved strategies through pharmacological therapies or catheter ablation. Additionally, patients with AF, who inherently have a worse prognosis as compared with those in sinus rhythm, may receive more comprehensive care and closer follow-up, which could facilitate the implementation of CRT.^1^ Overall, no further interactions were observed between AF and patient characteristics associated with CRT implantation beyond those with educational levels and concomitant valvular disease. In patients with AF, higher educational attainment was associated with increased likelihood of CRT implantation. Patients with lower educational attainment may face greater challenges in accessing and establishing contact with the healthcare system, in understanding the benefits and risks of CRT, in adhering to complex treatment regimens, and in effectively communicating with healthcare professionals. Such difficulties could be further exacerbated in the context of AF, where treatment decisions are even more complex.^22,23^ In patients without AF, the current study identified a lower likelihood of CRT implantation among those with valvular disease. Aortic valve disease represents the most common valvular heart disease among patients without AF and, if severe, may be prioritized in the clinical management pathway, potentially delaying CRT implantation.^24–26^

### Clinical outcomes

In this study, CRT was consistently associated with a lower risk of all-cause death, CV death, and first HF hospitalization, regardless of the presence of AF or subtype. One of the main challenges of CRT in patients with AF is achieving an adequate percentage of biventricular pacing, which can be particularly difficult in those with persistent or permanent AF.^27,28^ Over the years, atrioventricular node ablation has been investigated as a strategy to increase the percentage of effective biventricular pacing in patients with AF by abolishing physiologic atrioventricular conduction.^2,29^ Although no randomized studies have been performed to compare atrioventricular node ablation with medical rate control in achieving optimal biventricular pacing and improving clinical outcomes, several observational studies have consistently demonstrated a greater response to CRT among patients with AF who underwent atrioventricular node ablation.^2,29^ In our study, the association of CRT with better clinical outcomes remained consistent regardless of AF burden, as identified by AF type. Although with the inherent limitations of an observational study, this finding suggests that, in a contemporary real-world setting, adequate biventricular pacing may be hypothetically achieved in patients with AF through pharmacological therapy or AF ablation strategies other than atrioventricular node ablation, thereby maintaining the efficacy of CRT. The achievement of optimal biventricular pacing in this real-world cohort of patients receiving CRT may be further supported by the low proportion of patients who required atrioventricular node ablation during follow-up (i.e. approximately 9% in the CRT group), but this can only be speculated given the lack of a direct measurement. The association between CRT use and lower risk of adverse clinical events remained consistent in the cross-over analysis, except for outcomes related to HF hospitalizations. This may be explained by many CRT implants in the control group being preceded by an episode of HF hospitalization. Therefore, excluding these patients from the cross-over sensitivity analysis considerably reduced the number of events, thereby affecting statistical power. Of note, patients with CRT typically undergo more rigorous follow-up, often including telemonitoring, and CRT itself may facilitate the optimization of guideline-directed medical therapy.^30–32^ These factors could also partially explain the association between CRT use and better clinical outcomes observed in our study, beyond the delivery of effective resynchronization itself.^30–32^

### Limitations

Our study has limitations that are in need of acknowledgment when interpreting its results. First, as this is an observational study, causality cannot be established. Although extensive adjustments were performed and no association was observed in the falsification analysis, residual confounding cannot be fully ruled out. Additionally, data on the percentage of biventricular pacing were not available. Therefore, whether the lack of any modifier role for AF on the association between CRT and better clinical outcome is attributable to optimal biventricular pacing remains speculative. The classification of AF subtypes should be interpreted with caution, as these categories are not fixed and may change over time, with patients potentially transitioning from one subtype to another during follow-up.^33^ In patients who underwent CRT implantation, the variables from SwedeHF may not fully reflect baseline characteristics, as some data were recorded after implantation; however, all information was entered at most within one year of the procedure. The European Atlas of Cardiac Rhythm Disorders has recently highlighted substantial heterogeneity in procedural rates across Europe, with higher rates observed in high-income countries in Western and Northern Europe. Further studies from both European and non-European countries are therefore warranted to validate our findings.^34^ Finally, patients recruited into SwedeHF exhibit a lower comorbidity burden and better outcomes than the broader Swedish HF population.^35^ Therefore, the generalizability of our findings to a less selected, more comorbid HF cohort requires validation in further studies.

## Conclusions

In this nationwide contemporary real-world cohort of patients with HFrEF, CRT implantation rates remained stable at approximately one-quarter of the eligible population, regardless of history of or concomitant AF. CRT use was associated with a significant better prognosis, including lower risk of all-cause and CV death, and first HF hospitalization, independently of both the presence and subtype of AF. These associations should be interpreted with caution, and no conclusions regarding causality can be drawn, given the absence of data on biventricular pacing, the lack of randomization, and the potential for unmeasured confounding.

## Supplementary Material

euaf296_Supplementary_Data

## Data Availability

The data that support the findings of this study are available from the corresponding author, provided that data sharing is permitted by the European Union General Data Protection Regulation and appropriate ethics committees.
