# Supplementary material for: Associations between cardiac resynchronization therapy and clinical outcomes according to the atrial fibrillation status in patients with heart failure with reduced ejection fraction
Source: Europace. 2025 Nov 18;27(12):euaf296. doi: 10.1093/europace/euaf296 (PMC12678164; doi:10.1093/europace/euaf296)
Supplement: euaf296_Supplementary_Data [file euaf296_supplementary_data.docx]

**Supplementary Material**

**Table of contents**

**Supplementary Table S1. Variable definitions and sources.**

**Supplementary Table S2. Standardized mean differences at baseline and after overlap weighting.**

**Supplementary Table S3. Crude and using OW hazard ratios for overall population and according to AF status.**

**Supplementary Table S4. Multivariable hazard ratios for overall population.**

**Supplementary Table S5. Total number of HF hospitalizations (crude and using OW models).**

**Supplementary Table S6. Total number of HF hospitalizations (multivariable model).**

**Supplementary table S7. Subgroup analysis according to CRT type (multivariable model).**

**Supplementary Table S8. Subgroup analysis according to AF type (n = 2886) (OW model).**

**Supplementary Table S9. Subgroup analysis according to AF type (n = 2886) (multivariable model).**

**Supplementary Table S10. Number of HF hospitalizations according to AF type (n = 2886) (crude, OW, and multivariable models).**

**Supplementary Table S11. Baseline characteristics excluding patients with CRT implantation during follow-up (n = 2833).**

**Supplementary Table S12. Cross-over sensitivity analysis (crude and OW models).**

**Supplementary Table S13. Cross-over sensitivity analysis (multivariable model).**

**Supplementary Table S14. Sensitivity analysis including patients with index year equal or greater than 2017 (crude and OW models).**

**Supplementary Table S15. Sensitivity analysis including patients with index year equal or greater than 2017 (multivariable model).**

**Supplementary Table S16. Sensitivity analysis including patients with index year equal or greater than 2017 for the total number of HF hospitalizations (crude, OW, and multivariable models).**

**Supplementary Table S17. Sensitivity analysis considering AF as an electrocardiographic diagnosis at the time of registration in SwedeHF (multivariable and OW models).**

**Supplementary Table S18. Sensitivity analysis considering AF as an electrocardiographic diagnosis at the time of registration in SwedeHF or as a comorbidity diagnosis within one year prior to the index date (multivariable and OW models).**

**Supplementary Table S19. Sensitivity analysis excluding patients who underwent His ablation during follow-up (n = 3284).**

**Supplementary Table S20. Sensitivity analysis excluding AF patients who underwent pulmonary vein isolation during follow-up (n = 3464).**

**Supplementary Table S21. Falsification analysis (crude and OW models).**

**Supplementary Table S22. Falsification analysis (multivariable model).**

**Supplementary Figure S1. Study flowchart of patient selection.**

**Supplementary Figure S2.** **Propensity scores distribution in the overall and weighting populations.**

**Supplementary Table S1. Variable definitions and sources.**

| **Variable** | **Data source** | **Time period** |
| --- | --- | --- |
| **Exposure** | | |
| CRT and CRT type | ICD & PM Registry | Implantation |
| **Baseline characteristics** | | |
| Index year | SwedeHF/ ICD & PM Registry | Baseline |
| Sex | SwedeHF/ ICD & PM Registry | Baseline |
| Age | SwedeHF/ ICD & PM Registry | Baseline |
| QRS duration | SwedeHF | Registration |
| LBBB | SwedeHF | Registration |
| EF | SwedeHF | Registration |
| HF duration | NPR | No time restriction |
| Follow-up location | SwedeHF | Registration |
| Follow-up HF unit | SwedeHF | Registration |
| NYHA class | SwedeHF | Registration |
| SBP | SwedeHF | Registration |
| DBP | SwedeHF | Registration |
| MBP | SwedeHF | Registration |
| HR | SwedeHF | Registration |
| eGFR | SwedeHF | Registration |
| NT-pro BNP | SwedeHF | Registration |
| RASi/ARNI | NPDR: ATC C09A, C09B, C09C, C09D | Between 4 months before and 14 days after baseline |
| BB | NPDR: ATC C07 | Between 4 months before and 14 days after baseline |
| MRA | NPDR: ATC C03DA | Between 4 months before and 14 days after baseline |
| Loop diuretic | NPDR: ATC C03C | Between 4 months before and 14 days after baseline |
| SGLT2i | NPDR: ATC A10BK, A10BD16, A10BD15, A10BD20, A10BD23, A10BD27, A10BD25, A10BD21, A10BD29, A10BD24 | Between 4 months before and 14 days after baseline |
| CCB | NPDR: ATC C08 | Between 4 months before and 14 days after baseline |
| Antiplatelet | NPDR: ATC B01AC | Between 4 months before and 14 days after baseline |
| Anticoagulant | NPDR: ATC B01AA, B01AB, B01AE, B01AF, B01AX05 | Between 4 months before and 14 days after baseline |
| Insulin | NPDR: ATC A10A | Between 4 months before and 14 days after baseline |
| Oral antidiabetic | NPDR: ATC A10B | Between 4 months before and 14 days after baseline |
| Lipid lowering | NPDR: ATC C10 | Between 4 months before and 14 days after baseline |
| Digoxin | NPDR: ATC C01AA05 | Between 4 months before and 14 days after baseline |
| Nitrate | NPDR: ATC C01DA | Between 4 months before and 14 days after baseline |
| Antiarrhythmic | NPDR: ATC C01BD01, C01BD07, C01BC04, C01BC03, C07AA07, C01BA03 | Between 4 months before and 14 days after baseline |
| ICD (control group) | ICD & PM Registry | Baseline |
| PM (control group) | ICD & PM Registry | Baseline |
| Prior ICD/PM (CRT group) | ICD & PM Registry | Baseline |
| Diabetes | NPR: ICD-10 E10-4 | Within 5 years |
| AF | NPR: ICD-9/10 427D, I48 and SwedeHF (electrocardiogram showing AF) | Within 5 years for ICD 10 and since 1987 for ICD 9; at registration for electrocardiogram |
| AF type | NPR: ICD-9/10 427D | Within 5 years for ICD 10 and since 1987 for ICD 9 |
| Ischemic heart disease | NPR: ICD-9/10 410-4, I20-5, Z951, Z955 Procedure FNA, FNB, FNC, FND, FNE, FNF, FNH, FNG | Within 5 years for ICD 10 and since 1987 for ICD 9 |
| Hypertension | NPR: ICD-10 I10-5 | Within 5 years |
| PAD | NPR: ICD-10 I70-3 | Within 5 years |
| PCI | NPR: Procedure FNG | Within 5 years |
| CABG | NPR: ICD-9/10 Z951, Z955 Procedure FNA, FNB, FNC, FND, FNE, FNF, FNH | Within 5 years for ICD 10 and since 1987 for ICD 9 |
| Stroke/TIA | NPR: ICD-9/10 430-4, 438, I60-4, I690-4 | Within 5 years for ICD 10 and since 1987 for ICD 9 |
| Valvular disease | NPR: ICD-10 I05-8, I34-9, Q22, Q230-3, Q230-3, Q235-9, Z952-4 | Within 5 years |
| Cancer within 3 years | NPR: ICD-10 C | Within 3 years |
| COPD | NPR: ICD-10 J40-4 | Within 5 years |
| Liver disease | NPR: ICD-10 B18, I85, I864, I982, K70, K710, K711, K713-7, K72-4, K760, K762-9 | Within 5 years |
| Dementia | NPR: ICD-10 F00-4, R54 | Within 5 years |
| Bleeding | NPR: ICD-10 S064, S065, S066, I850, I983, K226, K250, K252, K254, K256, K260, K262, K264, K266, K270, K272, K274, K276, K280, K284, K286, K290, K625, K661, K920, K921, K922, H431, N02, R04, R58, T810, D629 Procedure DR029 | Within 5 years |
| Muscoloskeletal diseases within 3 years | NPR: ICD-10 M | Within 3 years |
| Family type | LISA | From the year before baseline |
| Children | RTP | From the year before baseline |
| Education | LISA | From the year before baseline |
| Income | LISA | From the year before baseline |
| Pulmonary vein isolation | NPR: FPB32, DF003, DF001 | No time restriction applied |
| Electrical cardioversion | NPR: DF026, DF027 | No time restriction applied |
| **Outcomes** | | |
| HF hospitalization | NPR: ICD-10 I110, I130, I132, I255, I420, I423, I425, I426, I427, I428, I429, I43, I50, J81, K761, R570^a^ | **-** |
| Cardiovascular death | CDR: ICD-10 I, J81, K761, R570, G45 | **-** |
| All-cause death | CDR | **-** |
| Hospitalization for trauma | NPR: ICD-10 S, T0, T10-4^a^ | **-** |
| Hospitalization for genito-urinary tract infection | NPR: ICD-10 N10, N11, N12, N136, N30, N33, N34,  N370, N390, N41, N45, N481, N482, N49, N51, N7^a^ | **-** |

*^a^ Hospitalizations are derived from diagnoses in main position, in-patient care.*

*Abbreviations: AF, atrial fibrillation; ARNI, angiotensin receptor/neprilysin inhibitor; ATC, Anatomical Therapeutic Chemical classification; BB, beta-blocker; CABG, coronary artery bypass grafting; CCB, calcium channel blocker; CDR, Cause of Death Registry; COPD, chronic obstructive pulmonary disease; CRT, cardiac resynchronization therapy; DBP, diastolic blood pressure; EF, ejection fraction; eGFR, estimated glomerular filtration rate; HF, heart failure; HR, heart rate; ICD, implantable cardioverter-defibrillator; ICD-10-SE, International Statistical Classification of Diseases, 10th revision, Swedish version; LBBB, left bundle branch block; LISA, Longitudinal Integrated Database for Health Insurance and Labour Market Studies; MBP, mean blood pressure; MRA, mineralocorticoid receptor antagonist; NPDR, National Prescribed Drug Register; NPR, National Patient Register; NT-pro BNP, N-terminal pro b-type natriuretic peptide;* *NYHA, New York Heart Association classification; PAD, peripheral artery disease; PCI, percutaneous coronary intervention; PM, pace-maker; RASi, renin–angiotensin system inhibitor; RTP, Registry of Total Population; SBP, systolic blood pressure; SGLT2i, Sodium-Glucose Cotransporter 2 inhibitors; SwedeHF, Swedish heart failure; TIA, Transient ischemic attack.*

**Supplementary Table S2. Standardized mean differences at baseline and after overlap weighting.**

|  | **Baseline**  **(n = 3530)**  **CRT (n = 873)**  **no-CRT (n = 2657)** | **Weighting**  **(n= 2966)**  **CRT (n = 852)**  **no-CRT (n = 2114)** |
| --- | --- | --- |
| **Variables** | **Standardized mean difference (%)** | **Standardized mean difference (%)** |
| **Calendar year**  2018-2022 vs 2014-2017 | 8.3 | 0.2 |
| **AF** | 5 | 2.9 |
| **Follow-up HF unit** | 7.3 | 2 |
| **Follow-up location**  *- Primary care vs Hospital*  *- Other vs Hospital* | 47.7  11 | 4.5  1.9 |
| **Sex**  *-Male* | 1.9 | 1.1 |
| **Duration of HF ≤ median** | 0.5 | 0.4 |
| **EF ^χ^**  < 30% vs 30-39% | 14 | 4.1 |
| **LBBB** | 8.1 | 2.1 |
| **SBP** (mmHg) **>** 110 | 16.6 | 5.8 |
| **MBP** (mmHg) > 90 | 6.9 | 2.6 |
| **HR** (beats/min) > 70 | 3.1 | 0.8 |
| **eGFR** (mL/min/1.73m^2^) ≥ 60 | 8.1 | 0.6 |
| **NT-pro BNP** (pg/mL) > median | 4.7 | 1.9 |
| **NYHA class**  NYHA III-IV vs NYHA I-II | 0.4 | 1.7 |
| **Age ≥** 75 years | 23.9 | 5.4 |
| **Children** | 0.6 | 0.6 |
| **Family situation**  *Living alone* | 8.8 | 2.7 |
| **Education**  *-Secondary vs Compulsory school*  *-University vs Compulsory school* | 4.8  9.4 | 0.2  2.1 |
| **Income** > median | 14.8 | 2.6 |
| **Hypertension** | 4.9 | 1.9 |
| **Diabetes** | 0.5 | 0.5 |
| **Ischemic heart disease** | 1.1 | 0.5 |
| **PAD** | 3 | 0.8 |
| **Stroke/TIA** | 5.1 | 1 |
| **Valvular disease** | 7.3 | 1.4 |
| **COPD** | 0.5 | 0.1 |
| **Liver disease** | 1.6 | 0.7 |
| **Dementia** | 11.6 | 0.9 |
| **Malignant cancer within 3 years** | 14 | 2.8 |
| **Muscolo-skeletal/connective tissue diseases within 3 years** | 3 | 1.2 |
| **Bleeding** | 13.7 | 3.5 |
| **BB** | 4.3 | 0.4 |
| **RASi/ARNI** | 10.9 | 0.9 |
| **MRA** | 25 | 5.5 |
| **SGLT2i** | 8.1 | 1.5 |
| **Loop diuretic** | 12.3 | 2.5 |
| **Nitrates** | 7.6 | 1.5 |
| **Anti-platelet** | 7.3 | 1.7 |
| **Anti-coagulant** | 9.9 | 3 |
| **Lipid lowering** | 6 | 0.7 |
| **CCB** | 8.1 | 1.5 |
| **Insulin** | 0.2 | 0.2 |
| **Oral anti-diabetic** | 7.1 | 1.1 |
| **Digoxin** | 10.4 | 4.9 |
| **Anti-arrhythmic** | 19.7 | 7.4 |

*Abbreviations: AF, atrial fibrillation; ARNI, angiotensin receptor/neprilysin inhibitor; BB, beta-blocker; CABG, coronary artery bypass grafting; CCB, calcium channel blocker; COPD, chronic obstructive pulmonary disease; CRT, cardiac resynchronization therapy; DBP, diastolic blood pressure; EF, ejection fraction; eGFR, estimated glomerular filtration rate; HF, heart failure; HR, heart rate; ICD, implantable cardioverter-defibrillator; IQR, interquartile range; LBBB, left bundle branch block; MBP, mean blood pressure; MRA, mineralocorticoid receptor antagonist; ms, milliseconds; N, number; NT-pro BNP, N-terminal pro b-type natriuretic peptide; NYHA, New York Heart Association classification; PAD, peripheral artery disease; PCI, percutaneous coronary intervention; PM, pace-maker; RASi, renin–angiotensin system inhibitor; SBP, systolic blood pressure; SGLT2i, Sodium-Glucose Cotransporter 2 inhibitors; TIA, Transient ischemic attack.*

|  | **Overall** | | **Overall** | | **AF** | **No-AF** |  |
| --- | --- | --- | --- | --- | --- | --- | --- |
| **Outcomes** | **Crude HR (95% CI)** | **P value** | **OW-HR (95% CI)** | **P value** | **OW-HR (95% CI)** | **OW-HR (95% CI)** | **P _for interaction_** |
| **Time to first HFH/CV death** | 0.67 (0.60; 0.74) | **< 0.001** | 0.71 (0.64; 0.79) | **< 0.001** | 0.73 (0.63; 0.84) | 0.68 (0.57; 0.81) | 0.538 |
| **Time to first HFH** | 0.73 (0.65; 0.82) | **< 0.001** | 0.74 (0.66; 0.84) | **< 0.001** | 0.78 (0.67; 0.91) | 0.70 (0.58; 0.84) | 0.374 |
| **Time to CV death** | 0.59 (0.51; 0.68) | **< 0.001** | 0.69 (0.59; 0.81) | **< 0.001** | 0.68 (0.56; 0.83) | 0.71 (0.55; 0.92) | 0.825 |
| **Time to all-cause death** | 0.59 (0.53; 0.67) | **< 0.001** | 0.72 (0.64; 0.81) | **< 0.001** | 0.73 (0.63; 0.86) | 0.70 (0.57; 0.85) | 0.682 |

**Supplementary Table S3. Crude and using OW hazard ratios for overall population and according to AF status.**

*Abbreviations*

*AF, atrial fibrillation; CI, confidence interval; CV, cardiovascular; HFH; heart failure hospitalization, HR, hazard ratio; OW, overlap weighting*

**Supplementary Table S4. Multivariable hazard ratios for overall population.**

|  | **Overall** | | **AF** | **No-AF** |  |
| --- | --- | --- | --- | --- | --- |
| **Outcomes** | **Multivariable HR (95% CI)** | **P value** | **Multivariable HR (95% CI)** | **Multivariable HR (95% CI)** | **P _for interaction_** |
| **Time to first HFH/CV death** | 0.66 (0.57; 0.76) | **< 0.001** | 0.66 (0.56; 0.79) | 0.66 (0.54; 0.80) | 0.949 |
| **Time to first HFH** | 0.68 (0.58; 0.80) | **< 0.001** | 0.70 (0.58; 0.84) | 0.66 (0.53; 0.81) | 0.647 |
| **Time to CV death** | 0.64 (0.52; 0.78) | **< 0.001** | 0.63 (0.50; 0.79) | 0.66 (0.49; 0.88) | 0.758 |
| **Time to all-cause death** | 0.65 (0.56; 0.76) | **< 0.001** | 0.67 (0.55; 0.80) | 0.63 (0.50; 0.79) | 0.677 |

*Abbreviations*

*AF, atrial fibrillation; CI, confidence interval; CV, cardiovascular; HFH; heart failure hospitalization, HR, hazard ratio.*

**Supplementary Table S5. Total number of HF hospitalizations (crude and using OW models).**

|  | **Overall** | | **Overall** | | **AF** | **No-AF** |  |
| --- | --- | --- | --- | --- | --- | --- | --- |
| **Outcomes** | **Crude IRR (95% CI)** | **P value** | **OW IRR (95% CI)** | **P value** | **OW IRR (95% CI)** | **OW**  **IRR (95% CI)** | **P _for interaction_** |
| **Overall population** | 0.97 (0.83; 1.12) | 0.673 | 1.00 (0.83; 1.22) | 0.975 | 1.06 (0.83; 1.35) | 0.89 (0.67; 1.18) | 0.359 |
| **Cross-over sensitivity** | 0.93 (0.77; 1.12) | 0.433 | 1.02 (0.82; 1.26) | 0.872 | 1.04 (0.80; 1.36) | 0.94 (0.69; 1.30) | 0.646 |

*Abbreviations*

*AF, atrial fibrillation; CI, confidence interval; CRT-D, cardiac resynchronization therapy-defibrillator; CRT-P, cardiac resynchronization therapy, pacemaker; HF, heart failure; IRR, Incidence Rate Ratio; OW, overlap weighting*

**Supplementary Table S6. Total number of HF hospitalizations (multivariable models).**

|  | **Overall** | | **AF** | **No-AF** |  |
| --- | --- | --- | --- | --- | --- |
| **Outcomes** | **Multivariable IRR (95% CI)** | **P value** | **Multivariable IRR (95% CI)** | **Multivariable IRR (95% CI)** | **P _for interaction_** |
| **Overall population** | 0.85 (0.71; 1.02) | 0.076 | 0.91 (0.73; 1.14) | 0.78 (0.62; 0.98) | 0.269 |
| **Cross-over sensitivity** | 1.02 (0.82; 1.27) | 0.836 | 1.02 (0.77; 1.33) | 1.03 (0.78; 1.38) | 0.903 |
| ***CRT-P vs no-CRT***  ***CRT-D vs no-CRT*** | 0.82 (0.64; 1.05)  0.87 (0.71; 1.06) | 0.111  0.163 | 0.95 (0.70; 1.27)  0.90 (0.70; 1.16) | 0.60 (0.40; 0.90)  0.84 (0.66; 1.08) | 0.172 |

*Abbreviations*

*AF, atrial fibrillation; CI, confidence interval; CRT-D, cardiac resynchronization therapy-defibrillator; CRT-P, cardiac resynchronization therapy-pacemaker; HF, heart failure; IRR, Incidence Rate Ratio*

**Supplementary table S7. Subgroup analysis according to CRT type (multivariable model).**

|  | **Overall** | | **AF** | **No-AF** |  |
| --- | --- | --- | --- | --- | --- |
| **Outcomes** | **Multivariable HR (95% CI)** | **P value** | **Multivariable HR (95% CI)** | **Multivariable HR (95% CI)** | **P _for interaction_** |
| **Time to first HFH/CV death**  *CRT-P vs no-CRT*  *CRT-D vs no-CRT* | 0.72 (0.59; 0.87)  0.63 (0.54; 0.74) | **0.001**  **< 0.001** | 0.73 (0.58; 0.91)  0.62 (0.50; 0.76) | 0.70 (0.51; 0.95)  0.65 (0.52; 0.80) | 0.929 |
| **Time to first HFH**  *CRT-P vs no-CRT*  *CRT-D vs no-CRT* | 0.71 (0.57; 0.88)  0.67 (0.56; 0.80) | **0.002**  **< 0.001** | 0.75 (0.58; 0.96)  0.67 (0.53; 0.83) | 0.63 (0.44; 0.90)  0.67 (0.53; 0.84) | 0.700 |
| **Time to CV death**  *CRT-P vs no-CRT*  *CRT-D vs no-*  *CRT* | 0.71 (0.54; 0.94)  0.60 (0.48; 0.75) | **0.018**  **< 0.001** | 0.69 (0.50; 0.94)  0.58 (0.44; 0.77) | 0.79 (0.49; 1.26)  0.61 (0.45; 0.85) | 0.861 |
| **Time to all-cause death**  *CRT-P vs no-CRT*  *CRT-D vs no-CRT* | 0.74 (0.60; 0.92)  0.60 (0.50; 0.72) | **0.005**  **< 0.001** | 0.74 (0.59; 0.94)  0.61 (0.48; 0.76) | 0.74 (0.51; 1.07)  0.59 (0.46; 0.96) | 0.992 |

*Abbreviations*

*AF, atrial fibrillation; CI, confidence interval; CRT-D, cardiac resynchronization therapy-defibrillator; CRT-P, cardiac resynchronization therapy-pacemaker; CV, cardiovascular; HFH, heart failure hospitalization; HR, hazard ratio.*

**Supplementary Table S8. Subgroup analysis according to AF type (n = 2886) (OW model).**

|  | **Overall** | | **Permanent AF** | **Persistent AF** | **Paroxysmal AF** | **No-AF** |  |
| --- | --- | --- | --- | --- | --- | --- | --- |
| **Outcomes** | **OW HR (95% CI)** | **P value** | **OW HR (95% CI)** | **OW HR (95% CI)** | **OW HR (95% CI)** | **OW HR (95% CI)** | **P _for interaction_** |
| **Time to first HFH/CV death** | 0.73 (0.64; 0.82) | **< 0.001** | 0.76 (0.61; 0.95) | 0.75 (0.47; 1.20) | 0.79 (0.57; 1.08) | 0.68 (0.58; 0.81) | 0.823 |
| **Time to first HFH** | 0.76 (0.66; 0.86) | **< 0.001** | 0.79 (0.62; 1.01) | 0.73 (0.44; 1.21) | 0.89 (0.63; 1.26) | 0.70 (0.59; 0.84) | 0.644 |
| **Time to CV death** | 0.69 (0.58; 0.82) | **< 0.001** | 0.62 (0.46; 0.83) | 0.98 (0.47; 2.06) | 0.70 (0.45; 1.11) | 0.71 (0.55; 0.92) | 0.705 |
| **Time to all-cause death** | 0.71 (0.62; 0.82) | **< 0.001** | 0.67 (0.53; 0.85) | 0.80 (0.44; 1.45) | 0.79 (0.56; 1.13) | 0.70 (0.57; 0.85) | 0.862 |

*Abbreviations*

*AF, atrial fibrillation; CI, confidence interval; CV, cardiovascular; HHF, heart failure hospitalization; HR, hazard ratio; OW-overlap weighting.*

**Supplementary Table S9. Subgroup analysis according to AF type (n = 2886) (multivariable model).**

|  | **Overall** | | **Permanent AF** | **Persistent AF** | **Paroxysmal AF** | **No-AF** |  |
| --- | --- | --- | --- | --- | --- | --- | --- |
| **Outcomes** | **Multivariable HR (95% CI)** | **P value** | **Multivariable HR (95% CI)** | **Multivariable HR (95% CI)** | **Multivariable HR (95% CI)** | **Multivariable HR (95% CI)** | **P _for interaction_** |
| **Time to first HFH/CV death** | 0.69 (0.59; 0.81) | **< 0.001** | 0.77 (0.60; 0.98) | 0.58 (0.34; 0.99) | 0.67 (0.48; 0.93) | 0.67 (0.55; 0.82) | 0.684 |
| **Time to first HFH** | 0.70 (0.59; 0.83) | **< 0.001** | 0.76 (0.59; 0.99) | 0.53 (0.30; 0.95) | 0.75 (0.52; 1.08) | 0.67 (0.54; 0.83) | 0.611 |
| **Time to CV death** | 0.67 (0.54; 0.84) | **< 0.001** | 0.66 (0.48; 0.91) | 1.09 (0.50; 2.39) | 0.57 (0.35; 0.94) | 0.68 (0.51; 0.91) | 0.572 |
| **Time to all-cause death** | 0.68 (0.57; 0.80) | **< 0.001** | 0.71 (0.55; 0.92) | 0.89 (0.48; 1.68) | 0.58 (0.40; 0.84) | 0.66 (0.53; 0.83) | 0.634 |

*Abbreviations*

*AF, atrial fibrillation; CI, confidence interval; CV, cardiovascular; HHF, heart failure hospitalization; HR, hazard ratio.*

**Supplementary Table S10. Number of HF hospitalizations according to AF type (n = 2886) (OW and multivariable models).**

|  | **Overall** | | **Permanent AF** | **Persistent AF** | **Paroxysmal AF** | **No-AF** |  |
| --- | --- | --- | --- | --- | --- | --- | --- |
| **Outcomes** | **OW IRR (95% CI)** | **P value** | **OW IRR (95% CI)** | **OW IRR (95% CI)** | **OW IRR (95% CI)** | **OW IRR (95% CI)** | **P _for interaction_** |
| **Number of HF hospitalizations** | 0.99 (0.80; 1.24) | 0.986 | 0.87 (0.60; 1.28) | 0.94 (0.47; 1.89) | 1.69 (1.00; 2.82) | 0.90 (0.68; 1.20) | 0.183 |

|  | **Overall** | | **Permanent AF** | **Persistent AF** | **Paroxysmal AF** | **No-AF** |  |
| --- | --- | --- | --- | --- | --- | --- | --- |
| **Outcomes** | **Multivariable IRR (95% CI)** | **P value** | **Multivariable IRR (95% CI)** | **Multivariable IRR (95% CI)** | **Multivariable IRR (95% CI)** | **Multivariable IRR (95% CI)** | **P _for interaction_** |
| **Number of HF hospitalizations** | 0.85 (0.70; 1.02) | 0.087 | 0.73 (0.54; 1.01) | 0.71 (0.37; 1.36) | 1.52 (0.99; 2.34) | 0.79 (0.63; 0.99) | **0.019** |

*Abbreviations*

*AF, atrial fibrillation; CI, confidence interval; HF, heart failure; IRR, incidence rate ratio; OW, overlap weighting.*

**Supplementary Table S11. Baseline characteristics excluding patients with CRT implantation during follow-up (n = 2833).**

| **Variables** | **CRT ^χ^** | **No-CRT ^χ^** | **P value** |
| --- | --- | --- | --- |
| **N (%)** | 873 | 1960 | - |
| **AF (%) ^χ^** | 467 (53.5) | 1046 (53.4) | 0.950 |
| **CRT type**  CRT-D  CRT-P | 591 (67.7)  282 (32.3) | -  - | - |
| **Calendar year ^χ^**  2014-2017  2018-2022 | 397 (45.5)  476 (54.5) | 997 (50.9)  963 (49.1) | **0.008** |
| **Follow-up HF unit ^χ^** | 712 (84.4) | 1465 (78.5) | **< 0.001** |
| **Follow-up location ^χ^**  *- Hospital*  *- Primary care*  *- Other* | 818 (94.7)  36 (4.2)  10 (1.2) | 1399 (73.9)  443 (23.4)  51 (2.7) | **< 0.001** |
| **Sex ^χ^**  *-Male* | 668 (76.5) | 1516 (77.4) | **0.628** |
| **Duration of HF** (days) | 1912 (517-3885) | 1932 (688-3762) | 0.205 |
| **Duration of HF > median ^χ^** | 435 (49.8) | 980 (50) | 0.933 |
| **EF ^χ^**  < 30%  30-39% | 377 (43.2)  496 (56.8) | 1014 (51.7)  946 (48.3) | **< 0.001** |
| **QRS duration** (ms) | 150 (134-166) | 162 (154-172) | **< 0.001** |
| **QRS ≥ 150** (ms) **^χ^** | 354 (50.6) | 1960 (100) | **< 0.001** |
| **LBBB ^χ^** | 239 (68.5) | 1218 (68.2) | 0.906 |
| **SBP** (mmHg) | 117 (105-130) | 120 (110-135) | **< 0.001** |
| **SBP** (mmHg) **>** 110 **^χ^** | 509 (61.3) | 1329 (69.6) | **< 0.001** |
| **DBP** (mmHg) | 70 (62-80) | 70 (63-80) | 0.835 |
| **MBP** (mmHg) | 86.7 (78.3-94) | 88.3 (80-96.7) | **0.003** |
| **MBP** (mmHg) > 90 **^χ^** | 306 (36.9) | 786 (41.1) | **0.039** |
| **HR** (beats/min) | 70 (62-78) | 70 (61-80) | 0.879 |
| **HR** (beats/min) > 70 **^χ^** | 352 (43.9) | 905 (46.9) | 0.139 |
| **eGFR** (mL/min/1.73m^2^) | 64.5 (49.3-83) | 59.3 (44.6-79) | **< 0.001** |
| **eGFR** (mL/min/1.73m^2^) ≥ 60 **^χ^** | 468 (56.7) | 940 (48.7) | **< 0.001** |
| **NT-pro BNP** (pg/mL) | 1998 (782-4630) | 2410 (973-5967) | **0.001** |
| **NT-pro BNP** (pg/mL) > median **^χ^** | 312 (48.2) | 818 (53.7) | **0.020** |
| **NYHA class** **^χ^**  NYHA I-II  NYHA III-IV | 389 (55.7)  309 (44.3) | 840 (55)  687 (45) | 0.751 |
| **Prior PM/ICD implantation** | 123 (14.1) | - | - |
| **Age** | 73 (66-78) | 78 (71-84) | **< 0.001** |
| **Age ≥** 75 years **^χ^** | 394 (45.1) | 1236 (63.1) | **< 0.001** |
| **Children** **^χ^** | 735 (84.2) | 1636 (83.5) | 0.631 |
| **Family situation** **^χ^**  *Living alone* | 358 (41) | 931 (47.5) | **< 0.001** |
| **Education ^χ^**  *-University*  *-Secondary school*  *-Compulsory*  *school* | 185 (21.7)  372 (43.7)  295 (34.6) | 327 (17)  763 (39.5)  839 (43.5) | **< 0.001** |
| **Income** | 1800  (1474-2553) | 1665.5 (1394-2184) | **< 0.001** |
| **Income** > median **^χ^** | 485 (55.6) | 900 (45.9) | **< 0.001** |
| **Hypertension** **^χ^** | 549 (62.9) | 1289 (65.8) | 0.138 |
| **Diabetes** **^χ^** | 268 (30.7) | 602 (30.7) | 0.993 |
| **Ischemic heart disease** **^χ^** | 555 (63.6) | 1278 (65.2) | 0.402 |
| **PCI** | 259 (29.7) | 540 (27.6) | 0.248 |
| **CABG** | 331 (37.9) | 761 (38.8) | 0.645 |
| **PAD ^χ^** | 82 (9.4) | 202 (10.3) | 0.455 |
| **Stroke/TIA ^χ^** | 114 (13.1) | 322 (16.4) | **0.022** |
| **Valvular disease ^χ^** | 184 (21.1) | 488 (24.9) | **0.027** |
| **COPD ^χ^** | 109 (12.5) | 249 (12.7) | 0.872 |
| **Liver disease ^χ^** | 18 (2.1) | 50 (2.6) | 0.432 |
| **Dementia ^χ^** | 2 (0.2) | 30 (1.5) | **0.002** |
| **Malignant cancer within 3 years ^χ^** | 82 (9.4) | 297 (15.2) | **< 0.001** |
| **Muscolo-skeletal/connective tissue diseases within 3 years ^χ^** | 285 (32.7) | 638 (32.6) | 0.960 |
| **Bleeding ^χ^** | 128 (14.7) | 394 (20.1) | **0.001** |
| **ICD** | - | 227 (11.6) | - |
| **PM** | - | 251 (12.8) | - |
| **BB ^χ^** | 794 (90.6) | 1741 (88.8) | 0.089 |
| **RASi/ARNI ^χ^** | 818 (93.7) | 1744 (89) | **< 0.001** |
| **MRA ^χ^** | 619 (70.9) | 1116 (56.9) | **< 0.001** |
| **SGLT2i ^χ^** | 100 (11.5) | 189 (9.6) | 0.141 |
| **Loop diuretic ^χ^** | 545 (62.4) | 1369 (69.9) | **< 0.001** |
| **Nitrates ^χ^** | 177 (20.3) | 471 (24) | **0.028** |
| **Anti-platelet ^χ^** | 332 (38) | 818 (41.7) | 0.064 |
| **Anti-coagulant ^χ^** | 458 (52.5) | 949 (48.4) | **0.047** |
| **Lipid lowering ^χ^** | 539 (61.7) | 1129 (57.6) | **0.039** |
| **CCB ^χ^** | 74 (8.5) | 243 (12.4) | **0.002** |
| **Insulin ^χ^** | 121 (13.9) | 280 (14.3) | 0.764 |
| **Oral anti-diabetic ^χ^** | 234 (26.8) | 459 (23.4) | 0.053 |
| **Digoxin ^χ^** | 114 (13.1) | 203 (10.4) | **0.035** |
| **Anti-arrhythmic ^χ^** | 106 (12.1) | 112 (5.7) | **< 0.001** |

*Abbreviations: AF, atrial fibrillation; ARNI, angiotensin receptor/neprilysin inhibitor; BB, beta-blocker; CABG, coronary artery bypass grafting; CCB, calcium channel blocker; COPD, chronic obstructive pulmonary disease; CRT, cardiac resynchronization therapy; DBP, diastolic blood pressure; EF, ejection fraction; eGFR, estimated glomerular filtration rate; HF, heart failure; HR, heart rate; ICD, implantable cardioverter-defibrillator; LBBB, left bundle branch block; MBP, mean blood pressure; MRA, mineralocorticoid receptor antagonist; ms, milliseconds; N, number; NT-pro BNP, N-terminal pro b-type natriuretic peptide; NYHA, New York Heart Association classification; PAD, peripheral artery disease; PCI, percutaneous coronary intervention; PM, pace-maker; RASi, renin–angiotensin system inhibitor; SBP, systolic blood pressure; SGLT2i, Sodium-Glucose Cotransporter 2 inhibitors; TIA, Transient ischemic attack.*

|  | **Overall** | | **Overall** | | **AF** | **No-AF** |  |
| --- | --- | --- | --- | --- | --- | --- | --- |
| **Outcomes** | **Crude HR (95% CI)** | **P value** | **OW HR (95% CI)** | **P value** | **OW HR (95% CI)** | **OW HR (95% CI)** | **P _for interaction_** |
| **Time to first HFH/CV death** | 0.76 (0.67; 0.85) | **< 0.001** | 0.89 (0.79; 1.01) | 0.079 | 0.84 (0.71; 0.99) | 0.97 (0.80; 1.18) | 0.257 |
| **Time to first HFH** | 0.91 (0.80; 1.04) | 0.153 | 1.03 (0.89; 1.18) | 0.697 | 0.96 (0.80; 1.15) | 1.13 (0.91; 1.40) | 0.264 |
| **Time to CV death** | 0.45 (0.38; 0.53) | **< 0.001** | 0.56 (0.47; 0.66) | **< 0.001** | 0.56 (0.45; 0.70) | 0.55 (0.41; 0.73) | 0.905 |
| **Time to all-cause death** | 0.43 (0.38; 0.49) | **< 0.001** | 0.55 (0.48; 0.64) | **< 0.001** | 0.59 (0.49; 0.71) | 0.49 (0.39; 0.61) | 0.191 |

**Supplementary Table S12. Cross-over sensitivity analysis (crude and OW models).**

*Abbreviations*

*AF, atrial fibrillation; CI, confidence interval; CV, cardiovascular; HFH, heart failure hospitalization; HR, hazard ratio; OW-overlap weighting.*

**Supplementary Table S13. Cross-over sensitivity analysis (multivariable model).**

|  | **Overall** | | **AF** | **No-AF** |  |
| --- | --- | --- | --- | --- | --- |
| **Outcomes** | **Multivariable HR (95% CI)** | **P value** | **Multivariable HR (95% CI)** | **Multivariable HR (95% CI)** | **P _for interaction_** |
| **Time to first HFH/CV death** | 0.83 (0.70; 0.98) | **0.026** | 0.77 (0.63; 0.93) | 0.93 (0.75; 1.16) | 0.131 |
| **Time to first HFH** | 0.94 (0.78; 1.13) | 0.505 | 0.87 (0.70; 1.08) | 1.06 (0.82; 1.35) | 0.178 |
| **Time to CV death** | 0.50 (0.40; 0.62) | **< 0.001** | 0.49 (0.38; 0.64) | 0.50 (0.37; 0.69) | 0.921 |
| **Time to all-cause death** | 0.49 (0.41; 0.59) | **< 0.001** | 0.53 (0.43; 0.64) | 0.44 (0.34; 0.57) | 0.238 |

*Abbreviations*

*AF, atrial fibrillation; CI, confidence interval; CV, cardiovascular; HFH, heart failure hospitalization; HR, hazard ratio.*

**Supplementary Table S14. Sensitivity analysis including patients with index year equal or greater than 2017 (crude and OW models).**

|  | **Overall** | | **Overall** | | **AF** | **No-AF** |  |
| --- | --- | --- | --- | --- | --- | --- | --- |
| **Outcomes** | **Crude HR (95% CI)** | **P value** | **OW HR (95% CI)** | **P value** | **OW HR (95% CI)** | **OW HR (95% CI)** | **P _for interaction_** |
| **Time to first HFH/CV death** | 0.63 (0.55-0.73) | **< 0.001** | 0.67 (0.57-0.78) | **< 0.001** | 0.74 (0.61-0.90) | 0.58 (0.46-0.73) | 0.105 |
| **Time to first HFH** | 0.67 (0.58-0.79) | **< 0.001** | 0.69 (0.59-0.81) | **< 0.001** | 0.78 (0.63-0.96) | 0.60 (0.47-0.77) | 0.118 |
| **Time to CV death** | 0.58 (0.47-0.73) | **< 0.001** | 0.68 (0.54-0.86) | **0.001** | 0.74 (0.56-0.98) | 0.58 (0.38-0.88) | 0.355 |
| **Time to all-cause death** | 0.59 (0.50-0.70) | **< 0.001** | 0.68 (0.57-0.81) | **< 0.001** | 0.75 (0.60-0.94) | 0.57 (0.42-0.78) | 0.157 |

*Abbreviations*

*AF, atrial fibrillation; CI, confidence interval; CV, cardiovascular; HFH, heart failure hospitalization; HR, hazard ratio; OW, overlap weighting.*

**Supplementary Table S15. Sensitivity analysis including patients with index year equal or greater than 2017 (multivariable model).**

|  | **Overall** | | **AF** | **No-AF** |  |
| --- | --- | --- | --- | --- | --- |
| **Outcomes** | **Multivariable HR (95% CI)** | **P value** | **Multivariable HR (95% CI)** | **Multivariable HR (95% CI)** | **P _for interaction_** |
| **Time to first HFH/CV death** | 0.67 (0.55-0.81) | **< 0.001** | 0.75 (0.60-0.93) | 0.57 (0.44-0.74) | 0.072 |
| **Time to first HFH** | 0.68 (0.56-0.84) | **< 0.001** | 0.77 (0.61-0.98) | 0.57 (0.43-0.76) | 0.069 |
| **Time to CV death** | 0.73 (0.55-0.97) | **0.030** | 0.77 (0.56-1.07) | 0.65 (0.41-1.02) | 0.492 |
| **Time to all-cause death** | 0.68 (0.54-0.86) | **0.001** | 0.74 (0.57-0.95) | 0.59 (0.42-0.83) | 0.246 |

*Abbreviations*

*AF, atrial fibrillation; CI, confidence interval; CV, cardiovascular; HFH, heart failure hospitalization; HR, hazard ratio.*

**Supplementary Table S16. Sensitivity analysis including patients with index year equal or greater than 2017 for the total number of HF hospitalizations (crude, OW, and multivariable models).**

|  | **Overall** | | **AF** | **No-AF** |  |
| --- | --- | --- | --- | --- | --- |
| **Outcomes** | **Multivariable IRR (95% CI)** | **P value** | **Multivariable IRR (95% CI)** | **Multivariable**  **IRR (95% CI)** | **P _for interaction_** |
| **Overall population** | 0.86 (0.69-1.08) | 0.198 | 1.04 (0.79-1.36) | 0.68 (0.51-0.91) | **0.017** |

|  | **Overall** | | **Overall** | | **AF** | **No-AF** |  |
| --- | --- | --- | --- | --- | --- | --- | --- |
| **Outcomes** | **Crude IRR (95% CI)** | **P value** | **OW IRR (95% CI)** | **P value** | **OW IRR (95% CI)** | **OW**  **IRR (95% CI)** | **P _for interaction_** |
| **Overall population** | 0.98 (0.78-1.14) | 0.577 | 0.99 (0.77-1.27) | 0.927 | 1.15 (0.83-1.58) | 0.76 (0.54-1.07) | 0.084 |

*Abbreviations*

*AF, atrial fibrillation; CI, confidence interval; CV, cardiovascular; HF, heart failure; IRR, incidence rate ratio; OW, overlap weighting.*

**Supplementary Table S17. Sensitivity analysis considering AF as an electrocardiographic diagnosis at the time of registration in SwedeHF (multivariable and OW models).**

|  | **Overall** | | **AF** | **No-AF** |  |
| --- | --- | --- | --- | --- | --- |
| **Outcomes** | **Multivariable HR (95% CI)** | **P value** | **Multivariable HR (95% CI)** | **Multivariable HR (95% CI)** | **P _for interaction_** |
| **Time to first HFH/CV death** | 0.67 (0.58; 0.79) | **< 0.001** | 0.70 (0.53; 0.92) | 0.67 (0.57; 0.79) | 0.741 |
| **Time to first HFH** | 0.69 (0.58; 0.81) | **< 0.001** | 0.73 (0.54; 0.97) | 0.68 (0.57; 0.81) | 0.666 |
| **Time to CV death** | 0.67 (0.54; 0.82) | **< 0.001** | 0.54 (0.37; 0.78) | 0.70 (0.56; 0.89) | 0.171 |
| **Time to all-cause death** | 0.66 (0.56; 0.78) | **< 0.001** | 0.60 (0.45; 0.78) | 0.68 (0.57; 0.82) | 0.381 |

|  | **Overall** | | **AF** | **No-AF** |  |
| --- | --- | --- | --- | --- | --- |
| **Outcome** | **Multivariable IRR (95% CI)** | **P value** | **Multivariable IRR (95% CI)** | **Multivariable IRR (95% CI)** | **P _for interaction_** |
| **Total number of HFH** | 0.88 (0.73; 1.06) | 0.167 | 0.70 (0.48; 1.01) | 0.92 (0.75; 1.11) | 0.151 |

|  | **Overall** | | **AF** | **No-AF** |  |
| --- | --- | --- | --- | --- | --- |
| **Outcomes** | **OW HR (95% CI)** | **P value** | **OW HR (95% CI)** | **OW HR (95% CI)** | **P _for interaction_** |
| **Time to first HFH/CV death** | 0.72 (0.64; 0.80) | **< 0.001** | 0.74 (0.58 ;0.95) | 0.71 (0.62 ;0.81) | 0.769 |
| **Time to first HFH** | 0.75 (0.66; 0.85) | **< 0.001** | 0.80 (0.61; 1.04) | 0.74 (0.64; 0.85) | 0.623 |
| **Time to CV death** | 0.70 (0.59; 0.82) | **< 0.001** | 0.59 (0.42; 0.83) | 0.73 (0.61; 0.89) | 0.279 |
| **Time to all-cause death** | 0.71 (0.63; 0.81) | **< 0.001** | 0.66 (0.50; 0.86) | 0.73 (0.63; 0.85) | 0.506 |

|  | **Overall** | | **AF** | **No-AF** |  |
| --- | --- | --- | --- | --- | --- |
| **Outcome** | **OW IRR (95% CI)** | **P value** | **OW IRR (95% CI)** | **OW IRR (95% CI)** | **P _for interaction_** |
| **Total number of HFH** | 1.02 (0.84; 1.25) | 0.812 | 0.78 (0.51; 1.17) | 1.09 (0.87; 1.37) | 0.159 |

*Abbreviations*

*AF, atrial fibrillation; CI, confidence interval; CV, cardiovascular; HFH, heart failure hospitalization; HR, hazard ratio; IRR, incidence rate ratio; OW, overlap weighting.*

**Supplementary Table S18. Sensitivity analysis considering AF as an electrocardiographic diagnosis at the time of registration in SwedeHF or as a comorbidity diagnosis within one year prior to the index date (multivariable and OW models).**

|  | **Overall** | | **AF** | **No-AF** |  |
| --- | --- | --- | --- | --- | --- |
| **Outcomes** | **Multivariable HR (95% CI)** | **P value** | **Multivariable HR (95% CI)** | **Multivariable HR (95% CI)** | **P _for interaction_** |
| **Time to first HFH/CV death** | 0.66 (0.57; 0.76) | **< 0.001** | 0.67 (0.56; 0.80) | 0.65 (0.54; 0.78) | 0.732 |
| **Time to first HFH** | 0.68 (0.58; 0.80) | **< 0.001** | 0.70 (0.58; 0.85) | 0.65 (0.53; 0.80) | 0.560 |
| **Time to CV death** | 0.64 (0.52: 0.78) | **< 0.001** | 0.63 (0.49; 0.80) | 0.66 (0.50; 0.86) | 0.753 |
| **Time to all-cause death** | 0.65 (0.56; 0.76) | **< 0.001** | 0.67 (0.56; 0.81) | 0.62 (0.50; 0.77) | 0.551 |

|  | **Overall** | | **AF** | **No-AF** |  |
| --- | --- | --- | --- | --- | --- |
| **Outcome** | **Multivariable IRR (95% CI)** | **P value** | **Multivariable IRR (95% CI)** | **Multivariable IRR (95% CI)** | **P _for interaction_** |
| **Total number of HFH** | 0.85 (0.71; 1.02) | 0.073 | 0.93 (0.74; 1.16) | 0.78 (0.62; 0.97) | 0.201 |

|  | **Overall** | | **AF** | **No-AF** |  |
| --- | --- | --- | --- | --- | --- |
| **Outcomes** | **OW HR (95% CI)** | **P value** | **OW HR (95% CI)** | **OW HR (95% CI)** | **P _for interaction_** |
| **Time to first HFH/CV death** | 0.71 (0.62; 0.79) | **< 0.001** | 0.73 (0.63; 0.85) | 0.68 (0.58; 0.80) | 0.573 |
| **Time to first HFH** | 0.74 (0.66; 0.84) | **< 0.001** | 0.78 (0.66; 0.91) | 0.71 (0.59; 0.84) | 0.436 |
| **Time to CV death** | 0.69 (0.59; 0.81) | **< 0.001** | 0.67 (0.55; 0.83) | 0.72 (0.56; 0.92) | 0.700 |
| **Time to all-cause death** | 0.72 (0.63: 0.81) | **< 0.001** | 0.73 (0.62; 0.87) | 0.69 (0.58; 0.84) | 0.671 |

|  | **Overall** | | **AF** | **No-AF** |  |
| --- | --- | --- | --- | --- | --- |
| **Outcome** | **OW IRR (95% CI)** | **P value** | **OW IRR (95% CI)** | **OW IRR (95% CI)** | **P _for interaction_** |
| **Total number of HFH** | 1.00 (0.83; 1.21) | 0.988 | 1.05 (0.82; 1.35) | 0.91 (0.69; 1.19) | 0.442 |

*Abbreviations*

*AF, atrial fibrillation; CI, confidence interval; CV, cardiovascular; HFH, heart failure hospitalization; HR, hazard ratio; IRR, incidence rate ratio; OW, overlap weighting.*

**Supplementary Table S19. Sensitivity analysis excluding patients who underwent His ablation during follow-up (n = 3284).**

|  | **Overall** | | **AF** | **No-AF** |  |
| --- | --- | --- | --- | --- | --- |
| **Outcomes** | **Multivariable HR (95% CI)** | **P value** | **Multivariable HR (95% CI)** | **Multivariable HR (95% CI)** | **P _for interaction_** |
| **Time to first HFH/CV death** | 0.67 (0.57; 0.78) | **< 0.001** | 0.67 (0.56; 0.80) | 0.66 (0.54; 0.81) | 0.861 |
| **Time to first HFH** | 0.70 (0.59; 0.82) | **< 0.001** | 0.72 (0.59; 0.87) | 0.66 (0.53; 0.83) | 0.507 |
| **Time to CV death** | 0.60 (0.49; 0.75) | **< 0.001** | 0.59 (0.46; 0.75) | 0.63 (0.47; 0.85) | 0.701 |
| **Time to all-cause death** | 0.62 (0.53; 0.73) | **< 0.001** | 0.63 (0.52; 0.76) | 0.61 (0.49; 0.77) | 0.846 |

|  | **Overall** | | **AF** | **No-AF** |  |
| --- | --- | --- | --- | --- | --- |
| **Outcome** | **Multivariable IRR (95% CI)** | **P value** | **Multivariable IRR (95% CI)** | **Multivariable IRR (95% CI)** | **P _for interaction_** |
| **Total number of HFH** | 0.85 (0.70; 1.02) | 0.081 | 0.91 (0.72; 1.15) | 0.78 (0.62; 0.99) | 0.304 |

|  | **Overall** | | **AF** | **No-AF** |  |
| --- | --- | --- | --- | --- | --- |
| **Outcomes** | **OW HR (95% CI)** | **P value** | **OW HR (95% CI)** | **OW HR (95% CI)** | **P _for interaction_** |
| **Time to first HFH/CV death** | 0.70 (0.63; 0.79) | **< 0.001** | 0.72 (0.61; 0.84) | 0.68 (0.57; 0.81) | 0.668 |
| **Time to first HFH** | 0.74 (0.65; 0.84) | **< 0.001** | 0.77 (0.65; 0.91) | 0.70 (0.58; 0.84) | 0.440 |
| **Time to CV death** | 0.68 (0.58; 0.80) | **< 0.001** | 0.67 (0.54; 0.82) | 0.70 (0.54; 0.91) | 0.776 |
| **Time to all-cause death** | 0.71 (0.62; 0.81) | **< 0.001** | 0.71 (0.60; 0.84) | 0.70 (0.57; 0.85) | 0.852 |

|  | **Overall** | | **AF** | **No-AF** |  |
| --- | --- | --- | --- | --- | --- |
| **Outcome** | **OW IRR (95% CI)** | **P value** | **OW IRR (95% CI)** | **OW IRR (95% CI)** | **P _for interaction_** |
| **Total number of HFH** | 1.02 (0.83; 1.24) | 0.881 | 1.06 (0.82; 1.37) | 0.92 (0.69; 1.23) | 0.474 |

*Abbreviations*

*AF, atrial fibrillation; CI, confidence interval; CV, cardiovascular; HFH, heart failure hospitalization; HR, hazard ratio; IRR, incidence rate ratio; OW, overlap weighting.*

**Supplementary Table S20. Sensitivity analysis excluding AF patients who underwent pulmonary vein isolation during follow-up (n = 3464).**

|  | **Overall** | | **AF** | **No-AF** |  |
| --- | --- | --- | --- | --- | --- |
| **Outcomes** | **Multivariable HR (95% CI)** | **P value** | **Multivariable HR (95% CI)** | **Multivariable HR (95% CI)** | **P _for interaction_** |
| **Time to first HFH/CV death** | 0.64 (0.56; 0.75) | **< 0.001** | 0.64 (0.54; 0.76) | 0.65 (0.53; 0.79) | 0.917 |
| **Time to first HFH** | 0.66 (0.56; 0.78) | **< 0.001** | 0.67 (0.56; 0.82) | 0.65 (0.52; 0.80) | 0.739 |
| **Time to CV death** | 0.64 (0.52; 0.78) | **< 0.001** | 0.63 (0.50; 0.80) | 0.66 (0.50; 0.88) | 0.764 |
| **Time to all-cause death** | 0.65 (0.55; 0.76) | **< 0.001** | 0.66 (0.55; 0.80) | 0.63 (0.50; 0.78) | 0.658 |

|  | **Overall** | | **AF** | **No-AF** |  |
| --- | --- | --- | --- | --- | --- |
| **Outcome** | **Multivariable IRR (95% CI)** | **P value** | **Multivariable IRR (95% CI)** | **Multivariable IRR (95% CI)** | **P _for interaction_** |
| **Total number of HFH** | 0.84 (0.70; 1.01) | 0.069 | 0.91 (0.72; 1.15) | 0.78 (0.62; 0.98) | 0.265 |

|  | **Overall** | | **AF** | **No-AF** |  |
| --- | --- | --- | --- | --- | --- |
| **Outcomes** | **OW HR (95% CI)** | **P value** | **OW HR (95% CI)** | **OW HR (95% CI)** | **P _for interaction_** |
| **Time to first HFH/CV death** | 0.70 (0.63; 0.78) | **< 0.001** | 0.71 (0.61; 0.83) | 0.68 (0.58; 0.81) | 0.717 |
| **Time to first HFH** | 0.74 (0.65; 0.83) | **< 0.001** | 0.76 (0.65; 0.90) | 0.70 (0.58; 0.84) | 0.488 |
| **Time to CV death** | 0.69 (0.59; 0.81) | **< 0.001** | 0.67 (0.55; 0.82) | 0.71 (0.55; 0.92) | 0.721 |
| **Time to all-cause death** | 0.71 (0.63; 0.81) | **< 0.001** | 0.72 (0.61; 0.85) | 0.70 (0.57; 0.85) | 0.817 |

|  | **Overall** | | **AF** | **No-AF** |  |
| --- | --- | --- | --- | --- | --- |
| **Outcome** | **OW IRR (95% CI)** | **P value** | **OW IRR (95% CI)** | **OW IRR (95% CI)** | **P _for interaction_** |
| **Total number of HFH** | 1.01 (0.83; 1.23) | 0.935 | 1.06 (0.83; 1.37) | 0.89 (0.67; 1.18) | 0.360 |

*Abbreviations*

*AF, atrial fibrillation; CI, confidence interval; CV, cardiovascular; HFH, heart failure hospitalization; HR, hazard ratio; IRR, incidence rate ratio; OW, overlap weighting.*

**Supplementary Table S21. Falsification analysis (crude and OW models).**

|  | **Overall** | | **Overall** | | **AF** | **No-AF** |  |
| --- | --- | --- | --- | --- | --- | --- | --- |
| **Outcome** | **Crude HR (95% CI)** | **P value** | **OW HR (95% CI)** | **P value** | **OW HR (95% CI)** | **OW HR (95% CI)** | **P _for interaction_** |
| **Time to first hospitalization for trauma or GUTI** | 0.75 (0.62; 0.91) | **0.003** | 0.92 (0.76; 1.12) | 0.425 | 0.89 (0.68; 1.15) | 0.97 (0.71; 1.31) | 0.675 |

*Abbreviations*

*AF, atrial fibrillation; CI, confidence interval; GUTI, genito-urinary tract infection; HR, hazard ratio; OW, overlap weighting*

**Supplementary Table S22. Falsification analysis (multivariable model).**

|  | **Overall** | | **AF** | **No-AF** |  |
| --- | --- | --- | --- | --- | --- |
| **Outcome** | **Multivariable HR (95% CI)** | **P value** | **Multivariable HR (95% CI)** | **Multivariable HR (95% CI)** | **P _for interaction_** |
| **Time to first hospitalization for trauma or GUTI** | 0.85 (0.66; 1.09) | 0.191 | 0.84 (0.62; 1.15) | 0.85 (0.61; 1.18) | 0.974 |

*Abbreviations*

*AF, atrial fibrillation; CI, confidence interval; GUTI, genito-urinary tract infection; HR, hazard ratio.*

**Supplementary Figure S1. Study flowchart of patient selection.**


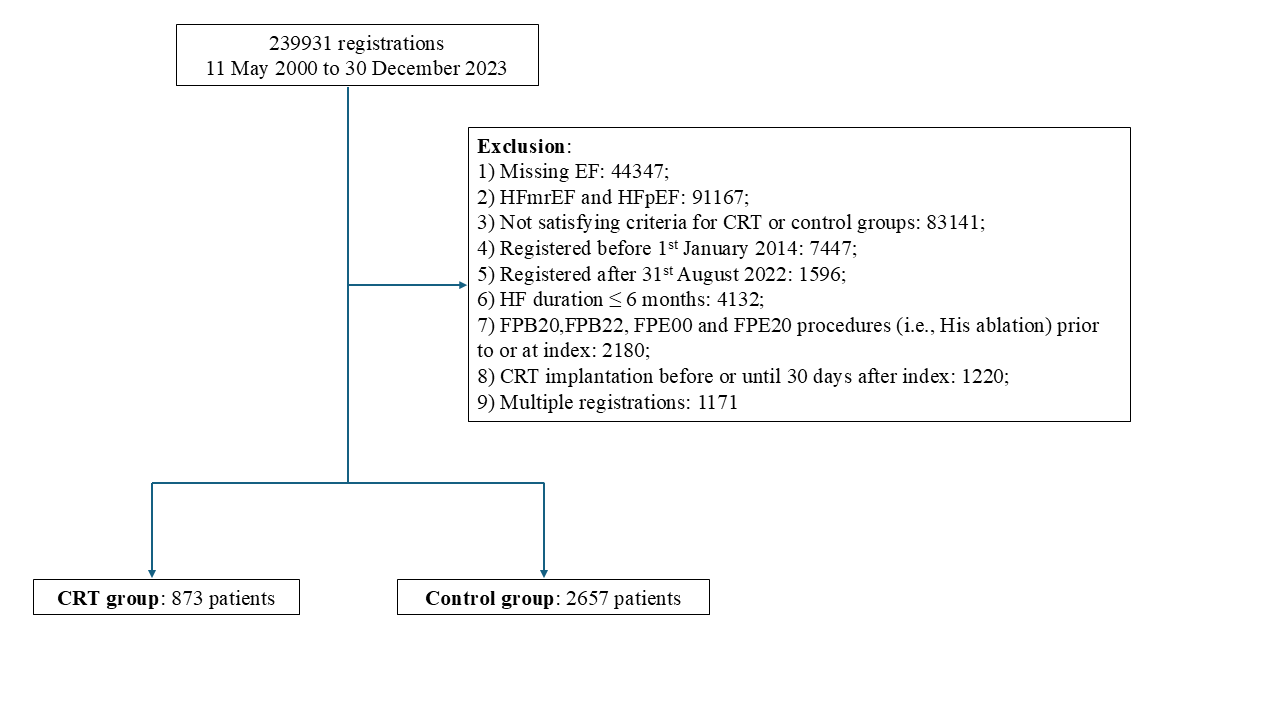


*Abbreviations*

*CRT, cardiac resynchronization therapy; EF, ejection fraction; HF, Heart Failure; HFmrEF, Heart Failure with mildly reduced Ejection Fraction; HFpEF, Heart Failure with preserved Ejection Fraction*

**Supplementary Figure S2. Propensity scores distribution in the overall and weighted populations.**


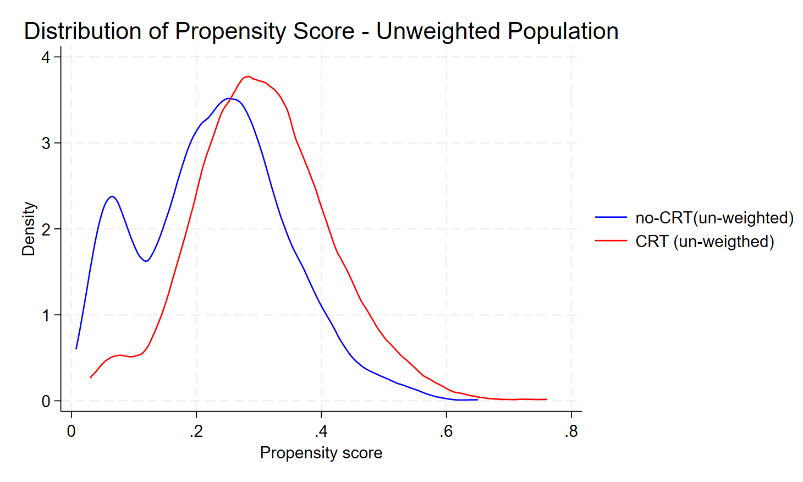


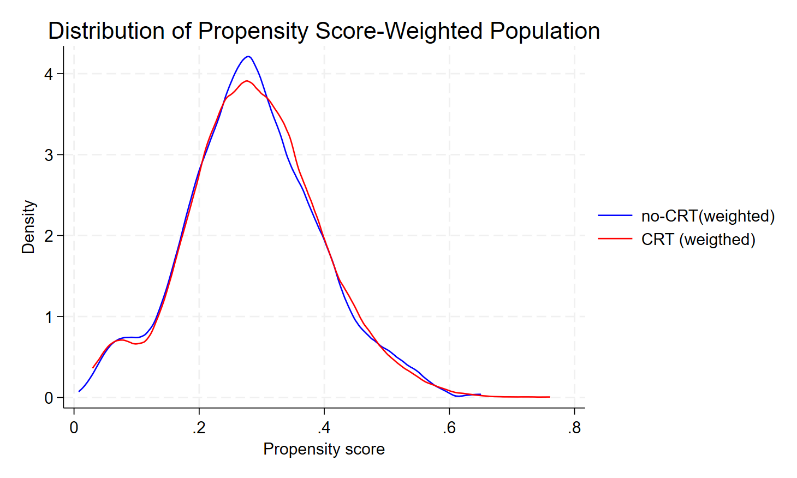


The distributions of propensity scores are displayed by Kernel density plots. After weighting, the distributions were nearly identical between the treatment and control groups, indicating improved covariate balance.

*Abbreviations: CRT, cardiac resynchronization therapy.*
